# Lipidomic characteristics and clinical findings of epileptic patients treated with valproic acid

**DOI:** 10.1111/jcmm.14464

**Published:** 2019-06-04

**Authors:** Rong Li, Xingyue Qin, Xiaoliu Liang, Meizhen Liu, Xiaoxi Zhang

**Affiliations:** ^1^ Guangxi Key Laboratory of Tumor Immunology and Microenvironmental Regulation Guilin Medical University Guilin PR China; ^2^ Department of Neurology (Area Two) Guigang City People's Hospital, The Eighth Affiliated Hospital of Guangxi Medical University Guigang PR China; ^3^ College of Pharmacy Guangxi Medical University Nanning PR China; ^4^ Center for Diabetic Systems Medicine, Guangxi Key Laboratory of Excellence Guilin Medical University Guilin China

**Keywords:** epilepsy, lipidome, metabolism, valproic acid

## Abstract

Our early study has found valproic acid (VPA)‐induced lipid dysmetabolism in animal model, however, the details of lipid profiling of VPA‐treated epileptic patients remain unknown. Therefore, in this study, the blood samples of VPA‐treated epileptic patients and VPA‐free controls were collected for lipidomic and biochemical assays. As results, clinical data showed the changes of some blood lipid molecules in VPA‐treated epileptic patients. In lipidomic assays, all 3797 annotated positive ions were identified prior to the data validation. In addition, the number of differentially expressed lipids were identified. And the 133 lipid molecules in VPA‐treated cases were significantly up‐regulated when compared to those in controls, while other 250 lipid metabolites were down‐regulated. Further, these lipid metabolites were mainly constituted with glycerolipids, glycerophopholipids, fatty acyls, sterol lipids. In addition, the most significant elevations of metabolite molecules of triglyceride, sphingomyelin, phosphorylcholine, ceramides, phenolic phthiocerol, as well as topped reductions of phosphoethanolamines, diradylglycerols, 1α,25‐dihydroxy‐24‐oxo‐22‐oxavitamin D3, 2‐deoxy‐20‐hydroxy‐5alpha‐ecdysone 3‐acetate, dolichyl‐4 phosphate were identified respectively. Taken together, these clinical findings demonstrate that negative impacts of exposure to VPA on expression of lipid mediators, progressively disrupting the functions of lipid molecules. Interestingly, these differentially expressed metabolites may be potential biomarkers for screening VPA‐induced dyslipidemia.

## 1 INTRODUCTION

In clinical observation, an antiepileptic medicine of valproic acid (VPA) may induce hepatic impairment in a time‐dependent manner, thus affecting metabolic functions as liver tissue is a vital endocrine organ.^1^ Increasing evidences indicate that chronic use of VPA are implicated in some adverse effects, such as gastrointestinal reaction, allergy, immunologic dysfunction.^2^ In addition, healthy risk of VPA on human weight gain is evident, and fatty acid translocase (CD36) and peroxisome proliferators‐activated receptor gama polymorphisms may be linked to VPA‐induced metabolic disorder.^3^ Accumulating evidences show that VPA may affect the blood contents of insulin, leptin, neuropeptide Y and ghrelin in epileptic children under insulin resistance, which the conditions are related to the development of obesity‐based metabolic disorders.^4^ Some clinical findings show VPA may induce elevations of blood lipids (triglycerides, free fatty acids), lipoproteins, and apolipoproteins in epileptic patients.^5^, ^6^ However, the clinical prophylaxis of VPA‐induced dyslipidemia is limited. In addition, detailed circulating lipid profiles in VPA‐treated epileptics remain unknown. Therefore, omics‐based tool and research can effectively provide mega experimental data before being revealed the promising findings of cellular components, functions, and molecular pathways, networks.^7^ Metabolome represents the complete profiling of molecule metabolites to be found in biological samples, such as liquid, cell and tissue.^8^ Lipidomics refers to a relatively hot tool that has been used to screen and identify species and functions of lipids in many metabolic diseases, such as obesity, diabetes, atherosclerosis, hyperlipemia.^9^ Therefore, in the current study, we reasoned that exposure to VPA may disrupt lipid metabolism to affect human endocrinological functions. To validate this hypothesis, we conducted molecular lipidomic tests and biochemical assays in blood samples between controls and VPA‐treated epileptics to identify differential expression of lipid metabolites, followed up revealing the biological functions and molecular pathways.

## 2 METHODS

### 2.1 Human sample handling

Three epileptic patients with seizures were medically imaged and diagnosed prior to being prescribed with VPA therapy (30 mg/day) for weeks. In clinical screening, other chronic diseases and metabolic disorders among these cases could be excluded through biochemical check‐up and clinical follow‐up. In addition, three adults with healthy conditions were set as a control group. Briefly, the blood samples of all subjects were isolated for plasma preparation. And parts of samples were used for lipidomic tests, while others were employed in instrument analysis and biological assays. The human protocols were implemented strictly on the basis of the Ethical Guidelines of the Declaration of Helsinki.^10^, ^11^

### 2.2 Blood VPA test with high performance liquid chromatography

Methodologically, the epileptic plasma samples were deproteinized by using commercially available reagents. To begin with, internal standards of VPA (>99%, Yuanye Biology, China) were prepared for different doses to plot a standard curve. The equal volumes of samples and standards were automatically added to a high performance liquid chromatography (HPLC) system (Shimadzu LC‐20A, Shimadzu, Japan) equipped with an SIL‐20AC detector and injector for VPA determination. The VPA separation was achieved by using an ASTON RG C18 column (5 μm, 4.6 × 50 mm, ANAX, China), followed by maintaining constant temperature 45°C in the column. In addition, the working mobile phase (acetonitrile:isopropanol:purified water = 23:8:69) was set as 1.1 mL/min flow rate. The VPA peak area was determined in comparisons of retention time (RT) of tested samples and internal standards. As results, the quantitative contents of plasma VPA were identified through a standard curve.^12^

### 2.3 Lipidomic analysis

Firstly, the plasma metabolites were extracted with 50% methanol buffer. After being centrifugated at 4,000 *g*, the supernatants were transferred to another 96‐well plate. All samples were obtained by the liquid chromatograph‐mass spectrometer (LC‐MS) system following machine orders. The chromatographic separation was conducted by using an ultra‐performance liquid chromatography system (SCIEX, UK). A chromatographic column (100 × 2.1 mm, 1.7 µm; Waters, UK) was applied to the reversed phase separation. The flow rate was maintained at 0.4 mL/min with mobile phase of solvent A and solvent B. A high‐resolution tandem mass spectrometer TripleTOF5600plus (SCIEX, UK) was employed to identify the metabolites form column. The data of mass spectrometry were collected following the IDA mode, and the TOF molecular weight ranged from 60 to 1200 Da. In data collection, the mass accuracy was calibrated every twenty samples. In addition, in an attempt to assess the LC‐MS stability, a quality control (QC) sample was identifiable after every ten samples.

### 2.4 Bioinformatic assay

Each positive ion was screened through pooling RT and mass charge ratio. Intensities of detectable peaks were recorded, and all information of a three dimensional matrix with peak indices and ion intensity was produced. The web‐accessible databases of Kyoto Encyclopedia of Genes and Genomes, Human Metabolome Database were employed to annotate the metabolites through correlating the accurate molecular mass data. If a mass difference of database value was <10 ppm, the identifiable metabolites would be annotated and the molecular formula would be further validated through the isotopic distribution determination. Additionally, an in‐house fragment spectrum library of metabolites was used to validate the identifiable metabolites. Those detectable features with <50% of QC samples or 80% of biological samples were excluded, other remaining peaks were evaluated with the k‐nearest neighbor algorithm to further enhance the quality of data. The principal components analysis (PCA) was implemented for outlier inspection and batch impacts assessment by using the pre‐processed dataset. Additionally, the relative standard deviations of the metabolic features were measured from total QC samples, and those data >30% were excluded. Wilcoxon test was performed to analyze differences in metabolite contents between 2 phenotypes. The *P*‐value was produced for multiple tests by using a false discovery rate (Benjamini‐Hochberg). Partial least squares‐discrimination analysis was assessed to differentiate the possible variables between groups. Further, a cut‐off value was applied in identification of all core features.

### 2.5 Statistical data

Statistical assessment was processed through statistical product and service solutions 19 (Chicago, IL). Fold‐change ratio of differentially expressed metabolites between two groups were analyzed by Student's *t* test. Groups were considered to be significantly different if a *P* < 0.05. Result was expressed as mean ± SD.^13^

## 3 RESULTS

### 3.1 Clinical images and blood data of VPA‐treated epileptic patients

**Figure 1:**
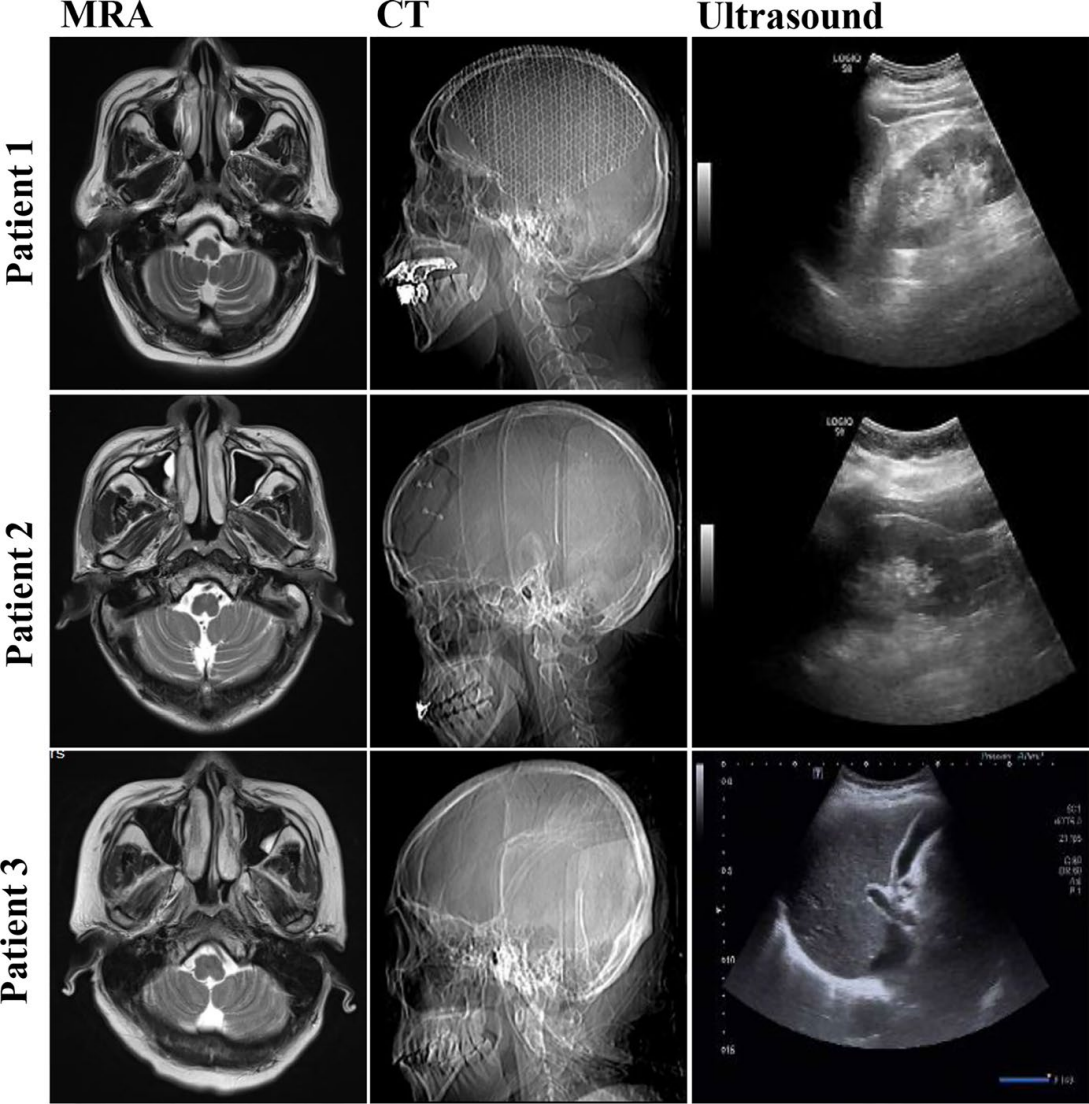
Preliminary medical imagines of valproic acid (VPA)‐treated epileptic patients. As shown in diagnostic images of magnetic resonance angiography (MRA) and computed tomography (CT), the VPA‐treated epileptics resulted in the brain impairment, mild leukoaraiosis, and brain atrophy. However, normal morphology and structure of other key internal organs of liver, spleen, kidney were detected in ultrasonic examination

In medical imaging inspection, these epileptic cases showed certain brain impairments, mild leukoaraiosis, brain atrophy. And other key internal organs (liver, spleen, kidney) with normal morphologies were observed in ultrasonic inspection (Figure 1). Results of HPLC analysis exhibited that blood VPA concentrations of epileptics were 54.13 ± 17.07 μg/mL following the treatment. As detailed in Table 1, a majority of diagnostic parameters were normal levels within the clinical ranges, including electrolyte, functional enzymes, and metabolic mediators. However, the contents of some blood lipids, such as TG, HDL‐C, Apo A1, Apo B, were altered when compared to the clinical references.

**Table 1:** The medically biochemical data of VPA‐treated epileptic patients

Parameters	Pooled data	Clinical ranges
Age (y)	51.0 ± 4.9	—
Sex (M/F)	2/1	—
K (mmol/L)	3.95 ± 0.48	3.5‐5.5
Na (mmol/L)	139.17 ± 4.73	135‐145
Cl (mmol/L)	101.80 ± 5.55	96‐108
Ca (mmol/L)	2.28 ± 0.12	2.1‐2.6
Mg (mmol/L)	0.91 ± 0.22	0.67‐1.04
P (mmol/L)	1.27 ± 0.2	0.96‐1.62
GLU (mmol/L)	7.19 ± 1.72	3.89‐6.11
AMY (U/L)	139 ± 48.54	<220
Urea (mmol/L)	3.71 ± 1.66	1.7‐8.3
Cr (μmol/L)	65 ± 18.36	44‐98
UA (μmol/L)	335.67 ± 94.77	150‐420
HCO_3_ (mmol/L)	25.13 ± 1.3	22‐28
CYS‐C (mg/L)	0.95 ± 0.26	0.55‐1.55
β2‐MG (mg/L)	1.46 ± 0.61	0‐3
CHO (mmol/L)	4.76 ± 0.74	3.12‐6.24
TG (mmol/L)	3.19 ± 0.21	<1.71
HDL‐C (mmol/L)	0.94 ± 0.23	0.91‐1.56
LDL‐C (mmol/L)	2.89 ± 0.78	<3.5
Apo A1 (g/L)	0.99 ± 0.29	1‐1.6
Apo B (g/L)	0.97 ± 0.09	0.6‐1.1
HsCRP (mg/L)	2.52 ± 2.27	<3
CK‐NAC (U/L)	41 ± 18.33	26‐174
CK‐MB (U/L)	17 ± 11.36	<24
LDH‐L (U/L)	184 ± 40.93	115‐220
α‐HBDH (U/L)	173.67 ± 54.86	72‐182
TBIL (μmol/L)	5.07 ± 1.75	3.4‐20.6
DBIL (μmol/L)	1.93 ± 0.85	<8.6
IBIL (μmol/L)	3.13 ± 0.9	<5.4
ALT (U/L)	12.33 ± 1.15	<35
AST (U/L)	16 ± 4.36	<40
ALP (U/L)	88 ± 6.24	40‐150
GGT (U/L)	50.33 ± 32.87	<32
TBA (μmol/L)	11.23 ± 5.91	<10
5‐NT (U/L)	2.23 ± 1	<10
TP (g/L)	64.73 ± 8.41	65‐85
ALB (g/L)	40.43 ± 1.92	40‐55
GLB (g/L)	24.30 ± 6.58	<45
AFP (ng/mL)	2.50 ± 1.47	<25
VPA (μg/mL)	54.13 ± 17.07	50‐100

Abbreviations: 5‐NT, 5‐nucleotide enzyme; AFP, alpha fetoprotein; ALB, albumin; ALP, alkaline phosphatase; ALT, glutamic‐pyruvic transaminase; AMY, amylase; Apo A1, apolipoprotein A1; Apo B, apolipoprotein B; AST, glutamic‐oxaloacetic transaminase; CHO, total cholesterol; CK‐MB, creatine kinase, MB form; CK‐NAC, creatine kinase; Cr, creatinine; CYS‐C, cystatin c; DBIL, direct bilirubin; dehydrogenase; F, female; GGT, glutathione transpeptidase; GLP, globulin; GLU, glucose in urine; HDL‐C, high‐density lipoprotein; HsCRP, hypersensitive C‐reactive protein; IBIL, indirect bilirubin; LDH‐L, lactate dehydrogenase L; LDL‐C, low‐density lipoprotein; M, male; TBA total, bile acids; TBIL, total bilirubin; TG, triglyceride; TP, total protein; UA, uric acid; VPA, valproate acid; α‐HBDH, α‐hydroxybutyrate; β2‐MG, β2‐microglobulin.

### 3.2 Clinical lipidomic characterization of VPA‐treated epileptic patients

**Figure 2:**
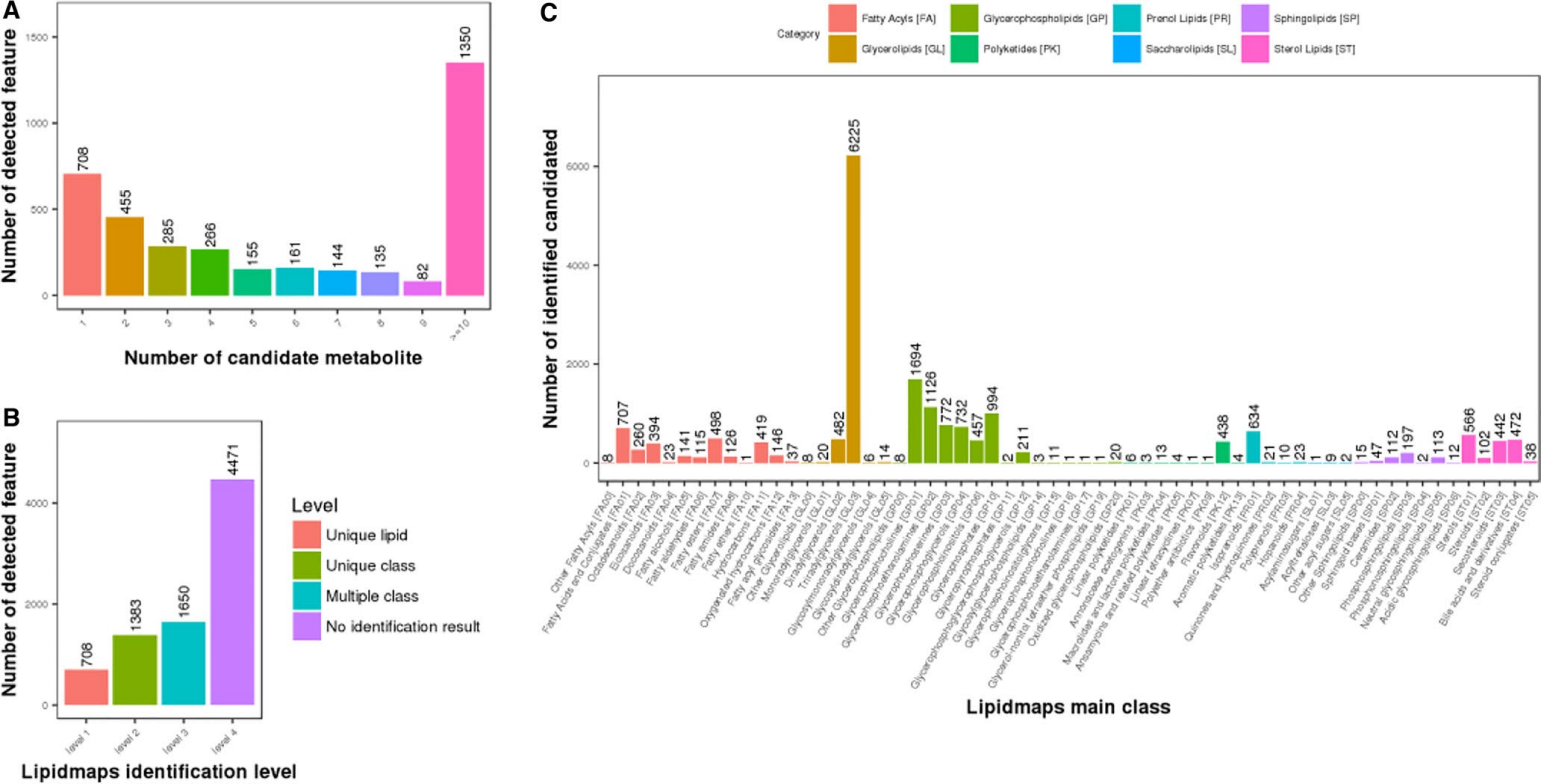
Clinical lipidomic characteristics of VPA‐treated epileptic patients. In total, all 3797 annotated positive ions were screened, and the candidate metabolites, lipidmaps identification level were numbered and classified (A,B). Further, lipidmaps main class was assorted following the degree (C). In metabolite quantitative statistics, 7700 high quality features from all 8212 features were identified

**Figure 3:**
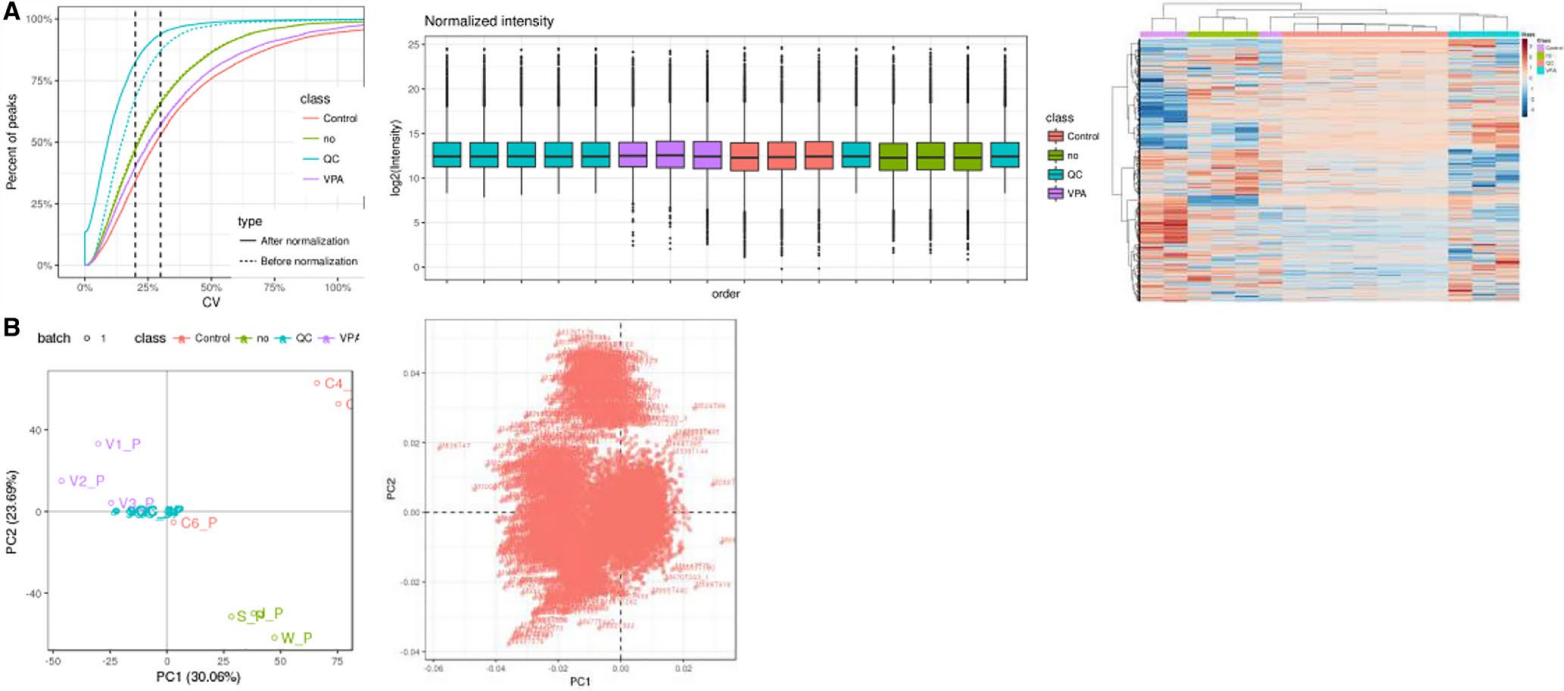
Validating data of valproic acid (VPA)‐treated epileptic patients. As shown in CV, lipid intensity distribution maps and heatmap, these metabolite molecules showed high repeatability of the samples tested (A). As revealed in principal components analysis score chart, the VPA samples exhibited dot‐based distribution trend and significant diversity of lipids (B)

In preliminary lipidomic statistics, a total of 3797 annotated positive ions were identified. And the number of candidate metabolite (Figure 2A), lipidmaps identification level (Figure 2B) were groped and listed respectively. Further, by use of identifiable metabolites to structure component category, lipidmaps main class was showed orderly (Figure 2C). In further metabolite quantitative statistics, high quality features of 7700 targets from total 8212 molecules were screened and identified. As showed in coefficient of variation, lipid intensity distribution maps, and heatmap, these identifiable targets showed high repeatability of the samples tested (Figure 3A). As revealed in score chart of PCA, the VPA‐treated samples showed in dot‐based distribution trend and significant diversity of lipids (Figure 3B), respectively.

### 3.3 The features of differential expressed lipids in VPA‐treated epileptic patients

**Figure 4:**
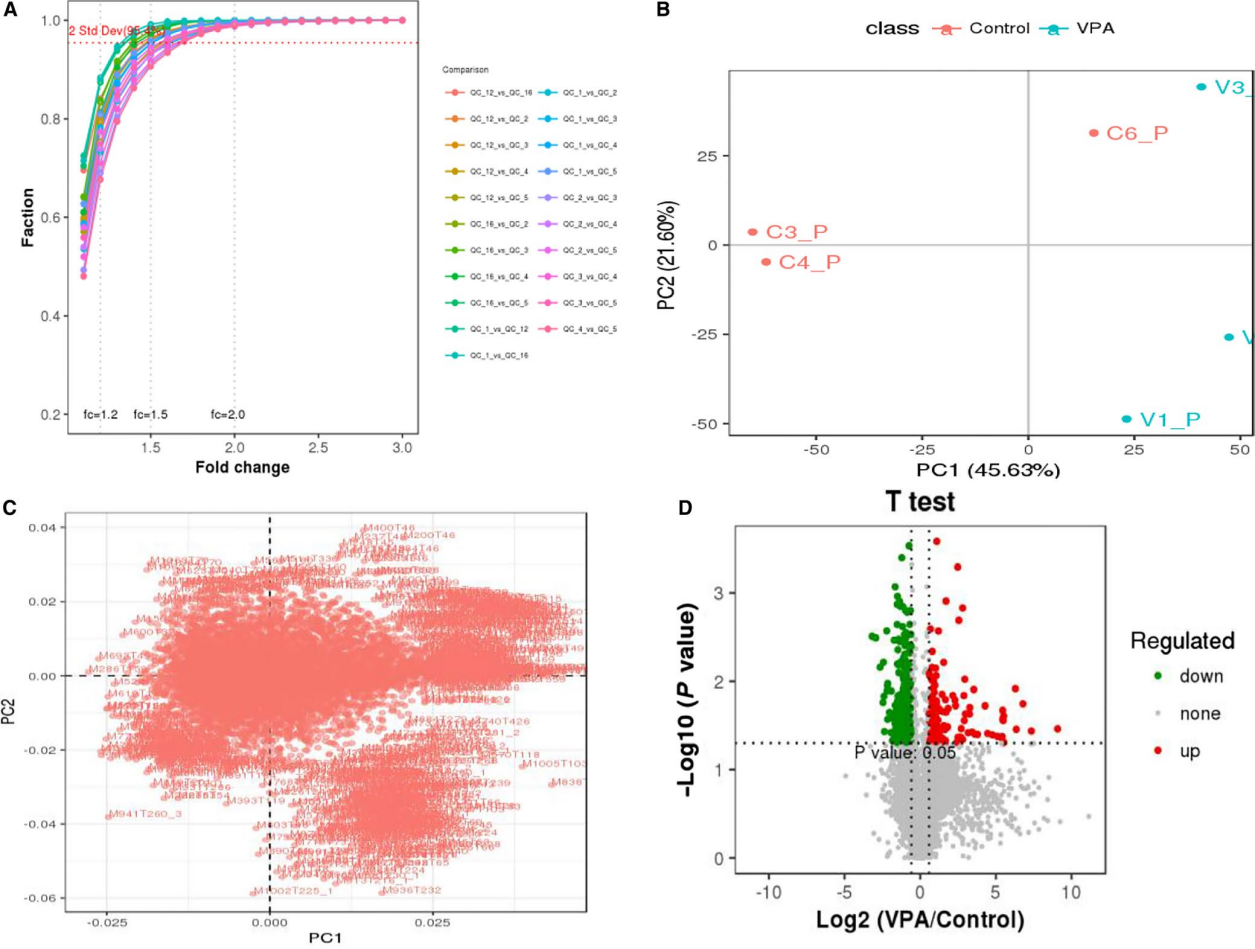
Differential expressed lipid molecules of valproic acid (VPA)‐treated epileptic patients. In ratio evaluation, less experimental errors in fold‐change of lipid percentage in all groups were observed (A). In principal components analysis (PCA), the trend of separation of VPA‐treated samples showed minor abnormal points and inconspicuous variability from the raw data (B). As shown in PCA scatter load diagram, the differential expressed lipids of VPA‐treated samples resulted in higher agminated class in comparison with those in controls (C). Followed by univariate analysis of fold‐change and p statistical test to obtain differential expressed lipids, 133 metabolites were significantly up‐regulated, while 250 molecules were down‐regulated (D)

To screen statistically and biologically significant lipid molecules from a large number of detectable metabolites, further analyses aimed to reveal the changes of metabolic processes and mechanisms following VPA treatment in epileptics. In ratio evaluation, relative low levels of experimental errors in fold‐change of lipids percentage in all groups were testified (Figure 4A). In PCA findings, the trend of lipid separation of VPA‐treated samples exhibited less abnormal points and inconspicuous variability from the original data (Figure 4B). As shown in PCA scatter load diagram, the differential expressed lipids of VPA‐treated samples resulted in higher agglomerative degree in comparison with those in controls (Figure 4C). After univariate analysis of fold‐change and p statistical test to obtain differentially expressed lipids, the identifiable 133 targets were significantly up‐regulated when compared to these in controls, while additional 250 molecules were down‐regulated markedly (Figure 4D). The details of all differential expressed lipid metabolites, including 133 up‐ and 250 down‐regulated molecules, were listed in Table S1.

## 4 DISCUSSION

Valproic acid, a medical antiepileptic, is found with induction of lipid‐associated dysmetabolism over long‐time treatment.^14^ Some evidences suggest that VPA may promote body weight gain and metabolic dysfunction in adolescent epilepsy.^15^ Pathogenically, certain genetic polymorphisms are likely related to the onset of VPA‐induced metabolic disorder,^16^ in which reports show the CYP2C19 polymorphism changes and VPA‐induced dysmetabolism in female patients with epilepsy.^17^ Pathologically, the potential metabolic dysregulation of VPA‐induced side‐effect should be detailed as revealed in the molecular mechanism. However, there are still limited literatures for this research topic, and then the full lipid characteristics in VPA‐treated epileptic patients remains unknown. Therefore, pursuit of promising biomolecules for screening lipid disorders may reduce VPA‐induced metabolic dysfunction. As detected in medical images, magnetic resonance angiography and computed tomography scans showed visible histopathologic injuries in brain tissues in these epileptics, suggesting brain injury might induce extra‐neural endocrine dysfunction. In clinical report, numerous unchanged blood parameters in VPA‐treated epileptics were showed, excluding chronic diseases and metabolic disorders associated with dysmetabolism. Instead, the levels of blood lipids molecules, including TG, HDL‐C, Apo A1, Apo B, were changed when compared to those in clinical references, indicating these epileptics might be in a trend of developing dyslipidemia. However, detailed metabolite profiling of VPA‐treated epileptics needs to be revealed.

Metabolomics refers to a scientific method for disclosing all biological fingerprints associated with metabolites, molecule intermediates, and products of metabolism.^18^ Metabolic profiling can provide a direct instantaneous snapshot of the physiology of targeting cell and organism.^19^ Lipidome is the entire components of cellular lipids, including the modifications made to an identifiable set of lipids from targeting cell and organism.^20^ Lipidomics represents the large‐scale assays for pathways and networks of cellular lipids in biological systems through conducting mass spectrometry techniques.^21^ Together, we aimed to use the lipidomic approach to disclose the all lipid metabolites in blood sample of VPA‐treated epileptic patients, followed by identification of the differential expressed lipids. As revealed in lipidmaps, a total of 3741 candidate metabolites were isolated and grouped as annotated features. As results, these lipid metabolites were mainly constituted with glycerolipids, glycerophopholipids, fatty acyls, sterol lipids. And the top expression of triradylglycerols was consistent with the significant elevation of blood TG content in VPA‐treated epileptics. In addition, some studies show that elevation in ether‐linked glycerolipids may be characteristic of human brain injury.^22^, ^23^ These findings indicated that increased lipid‐typed glycerolipids in VPA‐treated epileptics might be linked to the development of brain impairment, as validated in the medical images.

In further quantitative determination of these lipid metabolites, the higher quality features of 7700 lipids were identified, followed by quantitative QC. All testing methods showed the better credibility and repeatability of these identifiable data. In addition, in order to screen and characterize the differential expressed lipids of VPA‐treated epileptics, all testing lipid metabolites were further re‐assayed through designed criteria with fold‐change ratio higher than two and *P* < 0.05. Thus, top 133 up‐regulated and 250 down‐regulated lipid metabolites were identified. In addition, the detailed information of all these differential expressed lipids were listed. And the top 5 elevated metabolites of triglyceride, sphingomyelin, phosphorylcholine, ceramides, phenolic phthiocerol, as well as top 5 reduced metabolites of phosphoethanolamines, diradylglycerols, 1α,25‐dihydroxy‐24‐oxo‐22‐oxavitamin D3, 2‐deoxy‐20‐hydroxy‐5alpha‐ecdysone 3‐acetate, dolichyl‐4 phosphate were identified respectively. Taken together, we reasoned that top differential expressed lipids may contribute to development of alternative diagnosis for VPA‐induced metabolic disorders.

## 5 CONCLUSIONS

In brief, the clinical findings elucidate that VPA‐treated epileptic patients may induced brain impairment‐related dyslipidemia. Interestingly, lipidomic approach can contribute to identification of differential expressed lipid metabolites, and these molecules may be the potential markers for screening VPA‐induced dyslipidemia.

## CONFLICT OF INTEREST

The authors declare that there are no conflicts of interest.
