# Supplementary material for: Lipidomic characteristics and clinical findings of epileptic patients treated with valproic acid
Source: J Cell Mol Med. 2019 Jun 4;23(9):6017–23. doi: 10.1111/jcmm.14464 (PMC6714506; doi:10.1111/jcmm.14464)
Supplement: Supplementary file 1 [file JCMM-23-6017-s001.pdf]

ID	ratio	t.test_p.val	wilcox.test	t.test_p.val	wilcox.test	roc	lowROC	upROC
M1005T10	45.947	0.04929	0.1	0.593499	0.544554	1	1	1
M1007T24	0.502312	0.005709	0.1	0.593499	0.544554	1	1	1
M1018T40	1.768539	0.012081	0.1	0.593499	0.544554	1	1	1
M1030T80	0.537951	0.022197	0.1	0.593499	0.544554	1	1	1
M1051T39	0.655341	0.039162	0.1	0.593499	0.544554	1	1	1
M1064T11	5.609563	0.043189	0.1	0.593499	0.544554	1	1	1
M1068T21	9.691391	0.019873	0.1	0.593499	0.544554	1	1	1
M1086T69	0.222146	0.011246	0.1	0.593499	0.544554	1	1	1
M1087T69	0.181507	0.01743	0.1	0.593499	0.544554	1	1	1
M1121T11	27.36325	0.040431	0.1	0.593499	0.544554	1	1	1
M1150T27	0.571087	0.024205	0.1	0.593499	0.544554	1	1	1
M1159T29	1.902675	0.013293	0.1	0.593499	0.544554	1	1	1
M1160T29	2.1316	0.000261	0.1	0.593499	0.544554	1	1	1
M1164T27	1.615054	0.036908	0.1	0.593499	0.544554	1	1	1
M1230T25	9.006731	0.03818	0.1	0.593499	0.544554	1	1	1
M1426T23	0.189862	0.0148	0.1	0.593499	0.544554	1	1	1
M1466T29	0.400832	0.015467	0.1	0.593499	0.544554	1	1	1
M1469T29	0.451958	0.001423	0.1	0.593499	0.544554	1	1	1
M1475T27	0.603532	0.023306	0.1	0.593499	0.544554	1	1	1
M1475T29	0.218502	0.012839	0.1	0.593499	0.544554	1	1	1
M1482T23	0.224149	0.026988	0.1	0.593499	0.544554	1	1	1
M1486T26	2.095666	0.017222	0.1	0.593499	0.544554	1	1	1
M1502T29	0.483998	0.006942	0.1	0.593499	0.544554	1	1	1
M1505T23	0.110378	0.003094	0.1	0.593499	0.544554	1	1	1
M1509T25	0.40174	0.017917	0.1	0.593499	0.544554	1	1	1
M1510T25	0.392089	0.006187	0.1	0.593499	0.544554	1	1	1
M1511T25	0.567697	0.015116	0.1	0.593499	0.544554	1	1	1
M1513T26	0.616302	0.00877	0.1	0.593499	0.544554	1	1	1
M1561T24	0.193929	0.037034	0.1	0.593499	0.544554	1	1	1
M1571T32	0.612653	0.035039	0.1	0.593499	0.544554	1	1	1
M1596T32	0.49668	0.003945	0.1	0.593499	0.544554	1	1	1
M1600T34	0.558411	0.017186	0.1	0.593499	0.544554	1	1	1
M1612T25	0.225487	0.010578	0.1	0.593499	0.544554	1	1	1
M1613T25	0.284469	0.024931	0.1	0.593499	0.544554	1	1	1
M1614T25	0.303948	0.042529	0.1	0.593499	0.544554	1	1	1
M1621T29	0.46221	0.043625	0.1	0.593499	0.544554	1	1	1
M1627T34	0.460472	0.021997	0.1	0.593499	0.544554	1	1	1
M1629T28	0.52599	0.029043	0.1	0.593499	0.544554	1	1	1
M1636T25	0.255274	0.026092	0.1	0.593499	0.544554	1	1	1
M1650T34	0.555874	0.044335	0.1	0.593499	0.544554	1	1	1
M220T473	1.892892	0.040647	0.1	0.593499	0.544554	1	1	1
M282T320	0.441617	0.016709	0.1	0.593499	0.544554	1	1	1
M282T348	0.431493	0.021367	0.1	0.593499	0.544554	1	1	1
M310T143	1.576962	0.047448	0.1	0.593499	0.544554	1	1	1
M324T200	0.609165	0.029314	0.1	0.593499	0.544554	1	1	1
M326T186	0.457678	0.038704	0.1	0.593499	0.544554	1	1	1
M339T357	2.3509	0.033578	0.1	0.593499	0.544554	1	1	1
M341T60	1.757575	0.023206	0.1	0.593499	0.544554	1	1	1
M342T230	0.57428	0.039036	0.1	0.593499	0.544554	1	1	1
M343T231	0.605525	0.020207	0.1	0.593499	0.544554	1	1	1
M355T55	4.455121	0.014497	0.1	0.593499	0.544554	1	1	1
M361T70	0.461109	0.040523	0.1	0.593499	0.544554	1	1	1
M363T304	3.263292	0.02237	0.1	0.593499	0.544554	1	1	1
M363T327	0.620474	0.002266	0.1	0.593499	0.544554	1	1	1
M364T124	0.551013	0.028476	0.1	0.593499	0.544554	1	1	1
M365T48	3.589384	0.020366	0.1	0.593499	0.544554	1	1	1
M367T69	2.097913	0.01671	0.1	0.593499	0.544554	1	1	1

M369T452	0.130363	0.003223	0.1	0.593499	0.544554	1	1	1
M370T452	0.158741	0.006933	0.1	0.593499	0.544554	1	1	1
M385T67	0.406017	0.006335	0.1	0.593499	0.544554	1	1	1
M386T220	0.596045	0.008297	0.1	0.593499	0.544554	1	1	1
M390T69	0.641455	0.024954	0.1	0.593499	0.544554	1	1	1
M399T272	1.764638	0.045117	0.1	0.593499	0.544554	1	1	1
M401T99	0.367316	0.017556	0.1	0.593499	0.544554	1	1	1
M402T108	7.385541	0.014519	0.1	0.593499	0.544554	1	1	1
M407T89	0.640872	0.035277	0.1	0.593499	0.544554	1	1	1
M411T49	0.622128	0.040069	0.1	0.593499	0.544554	1	1	1
M412T279	2.037312	0.025186	0.1	0.593499	0.544554	1	1	1
M412T279	1.93212	0.02611	0.1	0.593499	0.544554	1	1	1
M422T46	81.39351	0.034959	0.1	0.593499	0.544554	1	1	1
M424T70	0.503388	0.034522	0.1	0.593499	0.544554	1	1	1
M425T70	0.573357	0.035125	0.1	0.593499	0.544554	1	1	1
M425T94	0.367141	0.046492	0.1	0.593499	0.544554	1	1	1
M426T309	4.896884	0.016325	0.1	0.593499	0.544554	1	1	1
M428T105	0.627521	0.02287	0.1	0.593499	0.544554	1	1	1
M428T46	5.571569	0.000509	0.1	0.593499	0.544554	1	1	1
M429T106	0.600362	0.022989	0.1	0.593499	0.544554	1	1	1
M433T131	1.553583	0.022498	0.1	0.593499	0.544554	1	1	1
M439T425	7.88944	0.023371	0.1	0.593499	0.544554	1	1	1
M444T128	2.713254	0.014077	0.1	0.593499	0.544554	1	1	1
M444T201	0.639079	0.04434	0.1	0.593499	0.544554	1	1	1
M447T121	0.243268	0.022475	0.1	0.593499	0.544554	1	1	1
M456T57	2.190471	0.046412	0.1	0.593499	0.544554	1	1	1
M467T136	0.552783	0.001658	0.1	0.593499	0.544554	1	1	1
M476T43	1.561817	0.045638	0.1	0.593499	0.544554	1	1	1
M480T143	0.659314	0.004014	0.1	0.593499	0.544554	1	1	1
M480T285	0.488946	0.027238	0.1	0.593499	0.544554	1	1	1
M480T78	2.420499	0.034116	0.1	0.593499	0.544554	1	1	1
M483T70	0.324045	0.026891	0.1	0.593499	0.544554	1	1	1
M484T70	0.383973	0.03455	0.1	0.593499	0.544554	1	1	1
M488T59	5.356131	0.049145	0.1	0.593499	0.544554	1	1	1
M492T112	0.352353	0.016745	0.1	0.593499	0.544554	1	1	1
M494T52	1.861601	0.049915	0.1	0.593499	0.544554	1	1	1
M495T454	6.971054	0.045677	0.1	0.593499	0.544554	1	1	1
M500T230	0.587258	0.048234	0.1	0.593499	0.544554	1	1	1
M501T223	0.590629	0.023008	0.1	0.593499	0.544554	1	1	1
M502T288	0.552739	0.041339	0.1	0.593499	0.544554	1	1	1
M502T70_	0.345123	0.021132	0.1	0.593499	0.544554	1	1	1
M503T70_	0.406746	0.037742	0.1	0.593499	0.544554	1	1	1
M512T123	0.603439	0.026167	0.1	0.593499	0.544554	1	1	1
M519T444	3.501917	0.032932	0.1	0.593499	0.544554	1	1	1
M519T70	0.403782	0.046527	0.1	0.593499	0.544554	1	1	1
M520T70_	0.317585	0.01867	0.1	0.593499	0.544554	1	1	1
M520T70_	0.324494	0.033925	0.1	0.593499	0.544554	1	1	1
M521T70	0.345015	0.03481	0.1	0.593499	0.544554	1	1	1
M522T296	1.598774	0.002559	0.1	0.593499	0.544554	1	1	1
M522T455	10.79288	0.037613	0.1	0.593499	0.544554	1	1	1
M522T69	0.382761	0.020471	0.1	0.593499	0.544554	1	1	1
M523T256	0.606894	0.011307	0.1	0.593499	0.544554	1	1	1
M526T67	0.369277	0.045554	0.1	0.593499	0.544554	1	1	1
M526T68	0.506375	0.027887	0.1	0.593499	0.544554	1	1	1
M527T282	0.445609	0.002403	0.1	0.593499	0.544554	1	1	1
M531T479	2.799277	0.044495	0.1	0.593499	0.544554	1	1	1
M531T66	0.481937	0.028862	0.1	0.593499	0.544554	1	1	1
M531T96	2.302112	0.002699	0.1	0.593499	0.544554	1	1	1

M535T321	0.531319	0.024972	0.1	0.593499	0.544554	1	1	1
M536T186	0.417871	0.021081	0.1	0.593499	0.544554	1	1	1
M539T159	0.399057	0.019685	0.1	0.593499	0.544554	1	1	1
M539T51	2.194877	0.046907	0.1	0.593499	0.544554	1	1	1
M540T256	0.464116	0.016414	0.1	0.593499	0.544554	1	1	1
M540T78	1.685906	0.020329	0.1	0.593499	0.544554	1	1	1
M542T159	2.126077	0.03557	0.1	0.593499	0.544554	1	1	1
M542T259	1.631632	0.016791	0.1	0.593499	0.544554	1	1	1
M542T70	0.315399	0.030069	0.1	0.593499	0.544554	1	1	1
M543T70	0.331924	0.027686	0.1	0.593499	0.544554	1	1	1
M544T68	0.434026	0.026424	0.1	0.593499	0.544554	1	1	1
M545T68	0.48844	0.02393	0.1	0.593499	0.544554	1	1	1
M548T68	0.49229	0.018358	0.1	0.593499	0.544554	1	1	1
M549T331	2.341016	0.042827	0.1	0.593499	0.544554	1	1	1
M550T66	0.409211	0.001418	0.1	0.593499	0.544554	1	1	1
M550T88	1.732986	0.004584	0.1	0.593499	0.544554	1	1	1
M551T258	0.496398	0.018466	0.1	0.593499	0.544554	1	1	1
M559T70	0.511674	0.024954	0.1	0.593499	0.544554	1	1	1
M560T122	0.656058	0.036528	0.1	0.593499	0.544554	1	1	1
M561T246	0.328693	0.035617	0.1	0.593499	0.544554	1	1	1
M562T132	0.361684	0.035442	0.1	0.593499	0.544554	1	1	1
M564T344	2.17781	0.039889	0.1	0.593499	0.544554	1	1	1
M564T499	1.743802	0.014891	0.1	0.593499	0.544554	1	1	1
M565T469	6.01537	0.042953	0.1	0.593499	0.544554	1	1	1
M565T470	0.533031	0.03434	0.1	0.593499	0.544554	1	1	1
M566T68	0.374937	0.007983	0.1	0.593499	0.544554	1	1	1
M567T68	0.369833	0.003341	0.1	0.593499	0.544554	1	1	1
M568T258	0.390402	0.043437	0.1	0.593499	0.544554	1	1	1
M568T66	0.317588	0.047922	0.1	0.593499	0.544554	1	1	1
M569T66	0.346148	0.019916	0.1	0.593499	0.544554	1	1	1
M570T108	1.71753	0.006965	0.1	0.593499	0.544554	1	1	1
M570T71	0.532305	0.019999	0.1	0.593499	0.544554	1	1	1
M571T71	0.632199	0.038236	0.1	0.593499	0.544554	1	1	1
M578T302	2.241179	0.046448	0.1	0.593499	0.544554	1	1	1
M584T387	1.86294	0.010196	0.1	0.593499	0.544554	1	1	1
M589T109	1.836628	0.044468	0.1	0.593499	0.544554	1	1	1
M589T353	0.645871	0.003896	0.1	0.593499	0.544554	1	1	1
M590T169	0.521511	0.019258	0.1	0.593499	0.544554	1	1	1
M590T332	1.701256	0.009693	0.1	0.593499	0.544554	1	1	1
M592T71	0.467533	0.032894	0.1	0.593499	0.544554	1	1	1
M595T441	1.622863	0.036805	0.1	0.593499	0.544554	1	1	1
M596T371	0.309158	0.024558	0.1	0.593499	0.544554	1	1	1
M597T87	0.567346	0.011269	0.1	0.593499	0.544554	1	1	1
M604T358	2.193003	0.049896	0.1	0.593499	0.544554	1	1	1
M605T381	1.538049	0.035555	0.1	0.593499	0.544554	1	1	1
M610T133	0.185561	0.006089	0.1	0.593499	0.544554	1	1	1
M615T88	2.921661	0.006104	0.1	0.593499	0.544554	1	1	1
M616T401	1.857899	0.045759	0.1	0.593499	0.544554	1	1	1
M619T363	0.429112	0.000399	0.1	0.593499	0.544554	1	1	1
M624T70	0.279233	0.046969	0.1	0.593499	0.544554	1	1	1
M628T160	0.600064	0.037394	0.1	0.593499	0.544554	1	1	1
M629T317	0.394785	0.001399	0.1	0.593499	0.544554	1	1	1
M631T337	0.489858	0.006985	0.1	0.593499	0.544554	1	1	1
M632T189	0.470659	0.040618	0.1	0.593499	0.544554	1	1	1
M632T481	5.709785	0.045114	0.1	0.593499	0.544554	1	1	1
M632T53	3.171898	0.01616	0.1	0.593499	0.544554	1	1	1
M638T314	0.435833	0.034992	0.1	0.593499	0.544554	1	1	1
M642T105	0.650855	0.034273	0.1	0.593499	0.544554	1	1	1

M644T160	0.578705	0.049398	0.1	0.593499	0.544554	1	1	1
M645T502	1.525407	0.027996	0.1	0.593499	0.544554	1	1	1
M648T519	3.254043	0.001236	0.1	0.593499	0.544554	1	1	1
M650T496	1.593602	0.028914	0.1	0.593499	0.544554	1	1	1
M652T310	1.885038	0.033837	0.1	0.593499	0.544554	1	1	1
M652T435	1.72381	0.031769	0.1	0.593499	0.544554	1	1	1
M655T156	1.726943	0.026219	0.1	0.593499	0.544554	1	1	1
M656T475	3.123356	0.033325	0.1	0.593499	0.544554	1	1	1
M667T341	0.410916	0.015812	0.1	0.593499	0.544554	1	1	1
M670T364	1.659862	0.015614	0.1	0.593499	0.544554	1	1	1
M670T393	2.85074	0.022031	0.1	0.593499	0.544554	1	1	1
M674T288	0.51923	0.033643	0.1	0.593499	0.544554	1	1	1
M682T382	1.941336	0.033964	0.1	0.593499	0.544554	1	1	1
M682T389	0.583614	0.007002	0.1	0.593499	0.544554	1	1	1
M684T230	0.540591	0.038977	0.1	0.593499	0.544554	1	1	1
M687T235	0.48545	0.033393	0.1	0.593499	0.544554	1	1	1
M687T488	2.420876	0.045528	0.1	0.593499	0.544554	1	1	1
M693T282	0.464469	0.015231	0.1	0.593499	0.544554	1	1	1
M698T474	1.770149	0.01792	0.1	0.593499	0.544554	1	1	1
M699T475	2.058142	0.016834	0.1	0.593499	0.544554	1	1	1
M705T287	0.624975	0.004773	0.1	0.593499	0.544554	1	1	1
M706T287	0.505065	0.012595	0.1	0.593499	0.544554	1	1	1
M709T259	0.569368	0.034947	0.1	0.593499	0.544554	1	1	1
M711T275	44.74693	0.027906	0.1	0.593499	0.544554	1	1	1
M711T303	0.399085	0.027816	0.1	0.593499	0.544554	1	1	1
M711T314	0.615439	0.008033	0.1	0.593499	0.544554	1	1	1
M712T264	0.535475	0.026046	0.1	0.593499	0.544554	1	1	1
M716T248	0.650356	0.00294	0.1	0.593499	0.544554	1	1	1
M717T201	5.05075	0.018926	0.1	0.593499	0.544554	1	1	1
M717T247	0.414555	0.044386	0.1	0.593499	0.544554	1	1	1
M718T283	0.48208	0.014015	0.1	0.593499	0.544554	1	1	1
M719T287	0.521149	0.021278	0.1	0.593499	0.544554	1	1	1
M719T303	2.387353	0.021429	0.1	0.593499	0.544554	1	1	1
M719T307	0.353107	0.001094	0.1	0.593499	0.544554	1	1	1
M720T277	43.62795	0.021346	0.1	0.593499	0.544554	1	1	1
M720T287	0.513622	0.003225	0.1	0.593499	0.544554	1	1	1
M720T303	2.101726	0.018141	0.1	0.593499	0.544554	1	1	1
M720T307	0.387838	0.003546	0.1	0.593499	0.544554	1	1	1
M721T230	0.498303	0.035808	0.1	0.593499	0.544554	1	1	1
M721T230	0.406498	0.032163	0.1	0.593499	0.544554	1	1	1
M721T312	0.465547	0.005416	0.1	0.593499	0.544554	1	1	1
M722T312	0.484347	0.002979	0.1	0.593499	0.544554	1	1	1
M723T312	0.470903	0.01573	0.1	0.593499	0.544554	1	1	1
M726T401	2.389016	0.049778	0.1	0.593499	0.544554	1	1	1
M728T238	0.351624	0.006362	0.1	0.593499	0.544554	1	1	1
M729T238	0.400817	0.0444	0.1	0.593499	0.544554	1	1	1
M729T291	0.507942	0.043286	0.1	0.593499	0.544554	1	1	1
M730T264	0.441636	0.036957	0.1	0.593499	0.544554	1	1	1
M730T282	0.511787	0.008017	0.1	0.593499	0.544554	1	1	1
M731T264	0.46476	0.028849	0.1	0.593499	0.544554	1	1	1
M732T264	0.473823	0.028668	0.1	0.593499	0.544554	1	1	1
M733T291	0.520711	0.045756	0.1	0.593499	0.544554	1	1	1
M734T57	6.971431	0.001479	0.1	0.593499	0.544554	1	1	1
M737T411	5.861296	0.00204	0.1	0.593499	0.544554	1	1	1
M738T484	2.240689	0.04078	0.1	0.593499	0.544554	1	1	1
M738T57	6.472234	0.043321	0.1	0.593499	0.544554	1	1	1
M739T484	2.080047	0.008602	0.1	0.593499	0.544554	1	1	1
M742T276	1.73623	0.028505	0.1	0.593499	0.544554	1	1	1

M743T287	0.407949	0.014022	0.1	0.593499	0.544554	1	1	1
M743T312	0.519872	0.002811	0.1	0.593499	0.544554	1	1	1
M744T287	0.433575	0.016432	0.1	0.593499	0.544554	1	1	1
M744T425	7.512844	0.027901	0.1	0.593499	0.544554	1	1	1
M744T499	2.023583	0.048053	0.1	0.593499	0.544554	1	1	1
M745T310	0.445252	0.036083	0.1	0.593499	0.544554	1	1	1
M746T310	0.460134	0.008927	0.1	0.593499	0.544554	1	1	1
M747T314	0.519617	0.007568	0.1	0.593499	0.544554	1	1	1
M748T315	0.531523	0.017508	0.1	0.593499	0.544554	1	1	1
M749T315	0.581325	0.010968	0.1	0.593499	0.544554	1	1	1
M750T237	0.498276	0.039703	0.1	0.593499	0.544554	1	1	1
M750T281	13.82299	0.039963	0.1	0.593499	0.544554	1	1	1
M750T314	0.61579	0.006231	0.1	0.593499	0.544554	1	1	1
M750T340	0.462464	0.012713	0.1	0.593499	0.544554	1	1	1
M750T400	1.962051	0.011186	0.1	0.593499	0.544554	1	1	1
M754T475	0.454254	0.039598	0.1	0.593499	0.544554	1	1	1
M756T334	1.819714	0.031274	0.1	0.593499	0.544554	1	1	1
M760T315	0.665162	0.015944	0.1	0.593499	0.544554	1	1	1
M765T287	0.626614	0.040007	0.1	0.593499	0.544554	1	1	1
M766T384	2.780331	0.048294	0.1	0.593499	0.544554	1	1	1
M766T393	1.511628	0.023224	0.1	0.593499	0.544554	1	1	1
M767T281	0.467103	0.02472	0.1	0.593499	0.544554	1	1	1
M768T281	0.498054	0.018323	0.1	0.593499	0.544554	1	1	1
M768T401	3.144032	0.034894	0.1	0.593499	0.544554	1	1	1
M769T258	0.55753	0.032244	0.1	0.593499	0.544554	1	1	1
M770T258	0.347891	0.002273	0.1	0.593499	0.544554	1	1	1
M772T316	0.435185	0.034148	0.1	0.593499	0.544554	1	1	1
M773T287	0.656683	0.038206	0.1	0.593499	0.544554	1	1	1
M773T320	0.491456	0.004971	0.1	0.593499	0.544554	1	1	1
M774T287	0.650735	0.049201	0.1	0.593499	0.544554	1	1	1
M774T320	0.508768	0.001576	0.1	0.593499	0.544554	1	1	1
M775T320	0.390279	0.001251	0.1	0.593499	0.544554	1	1	1
M775T323	0.634451	0.020128	0.1	0.593499	0.544554	1	1	1
M776T343	0.530155	0.00839	0.1	0.593499	0.544554	1	1	1
M780T226	0.498363	0.018675	0.1	0.593499	0.544554	1	1	1
M781T364	0.348256	0.001342	0.1	0.593499	0.544554	1	1	1
M781T426	5.96052	0.043758	0.1	0.593499	0.544554	1	1	1
M782T363	0.317132	0.000851	0.1	0.593499	0.544554	1	1	1
M784T358	0.577785	0.042922	0.1	0.593499	0.544554	1	1	1
M785T184	0.345581	0.048812	0.1	0.593499	0.544554	1	1	1
M785T352	0.591134	0.01154	0.1	0.593499	0.544554	1	1	1
M792T399	2.556018	0.046755	0.1	0.593499	0.544554	1	1	1
M793T237	0.408973	0.024087	0.1	0.593499	0.544554	1	1	1
M793T243	0.376078	0.038748	0.1	0.593499	0.544554	1	1	1
M793T284	0.50849	0.048462	0.1	0.593499	0.544554	1	1	1
M793T352	0.647724	0.044923	0.1	0.593499	0.544554	1	1	1
M793T433	2.07701	0.035042	0.1	0.593499	0.544554	1	1	1
M794T284	0.549801	0.038652	0.1	0.593499	0.544554	1	1	1
M794T352	0.533614	0.01456	0.1	0.593499	0.544554	1	1	1
M796T345	0.641253	0.040363	0.1	0.593499	0.544554	1	1	1
M796T374	0.540438	0.044673	0.1	0.593499	0.544554	1	1	1
M798T271	1.511959	0.008171	0.1	0.593499	0.544554	1	1	1
M799T316	0.498962	0.015102	0.1	0.593499	0.544554	1	1	1
M800T337	0.480152	0.008939	0.1	0.593499	0.544554	1	1	1
M800T454	6.533502	0.03445	0.1	0.593499	0.544554	1	1	1
M801T337	0.47705	0.011076	0.1	0.593499	0.544554	1	1	1
M802T213	0.38755	0.030044	0.1	0.593499	0.544554	1	1	1
M802T336	0.505095	0.030956	0.1	0.593499	0.544554	1	1	1

M803T336	0.524014	0.027652	0.1	0.593499	0.544554	1	1	1
M804T228	0.330521	0.021461	0.1	0.593499	0.544554	1	1	1
M804T258	0.35954	0.004404	0.1	0.593499	0.544554	1	1	1
M805T227	0.372313	0.006559	0.1	0.593499	0.544554	1	1	1
M807T258	0.359934	0.014592	0.1	0.593499	0.544554	1	1	1
M808T258	0.353764	0.022161	0.1	0.593499	0.544554	1	1	1
M811T296	0.597364	0.031769	0.1	0.593499	0.544554	1	1	1
M812T296	0.607952	0.02864	0.1	0.593499	0.544554	1	1	1
M812T323	0.495583	0.003453	0.1	0.593499	0.544554	1	1	1
M812T346	0.608391	0.004598	0.1	0.593499	0.544554	1	1	1
M813T323	0.523475	0.003733	0.1	0.593499	0.544554	1	1	1
M813T346	0.529668	0.01903	0.1	0.593499	0.544554	1	1	1
M814T323	0.567015	0.007449	0.1	0.593499	0.544554	1	1	1
M814T345	0.625519	0.029062	0.1	0.593499	0.544554	1	1	1
M815T323	0.540063	0.038997	0.1	0.593499	0.544554	1	1	1
M815T345	0.632074	0.024551	0.1	0.593499	0.544554	1	1	1
M816T345	0.644142	0.035555	0.1	0.593499	0.544554	1	1	1
M819T280	0.420761	0.025104	0.1	0.593499	0.544554	1	1	1
M820T315	0.638261	0.001577	0.1	0.593499	0.544554	1	1	1
M821T258	0.449346	0.023356	0.1	0.593499	0.544554	1	1	1
M821T363	0.303153	0.003417	0.1	0.593499	0.544554	1	1	1
M822T337	0.506817	0.027204	0.1	0.593499	0.544554	1	1	1
M823T337	0.491314	0.017119	0.1	0.593499	0.544554	1	1	1
M825T239	110.571	0.018019	0.1	0.593499	0.544554	1	1	1
M825T342	0.453318	0.046671	0.1	0.593499	0.544554	1	1	1
M828T224	0.261354	0.038097	0.1	0.593499	0.544554	1	1	1
M829T258	0.498016	0.015191	0.1	0.593499	0.544554	1	1	1
M830T258	0.488326	0.009821	0.1	0.593499	0.544554	1	1	1
M832T237	0.357686	0.048851	0.1	0.593499	0.544554	1	1	1
M832T333	0.65871	0.039759	0.1	0.593499	0.544554	1	1	1
M833T236	0.468881	0.035771	0.1	0.593499	0.544554	1	1	1
M833T333	0.623112	0.03269	0.1	0.593499	0.544554	1	1	1
M833T351	0.64566	0.010771	0.1	0.593499	0.544554	1	1	1
M834T323	0.591822	0.019277	0.1	0.593499	0.544554	1	1	1
M835T262	0.522232	0.045577	0.1	0.593499	0.544554	1	1	1
M835T323	0.594075	0.014624	0.1	0.593499	0.544554	1	1	1
M836T309	1.622053	0.045208	0.1	0.593499	0.544554	1	1	1
M836T373	0.634491	0.040705	0.1	0.593499	0.544554	1	1	1
M840T243	78.04586	0.01211	0.1	0.593499	0.544554	1	1	1
M840T348	0.589413	0.033085	0.1	0.593499	0.544554	1	1	1
M841T348	0.554411	0.0128	0.1	0.593499	0.544554	1	1	1
M846T256	0.541968	0.048919	0.1	0.593499	0.544554	1	1	1
M846T284	0.467362	0.009518	0.1	0.593499	0.544554	1	1	1
M847T322	2.24962	0.027566	0.1	0.593499	0.544554	1	1	1
M848T242	34.25883	0.042107	0.1	0.593499	0.544554	1	1	1
M849T319	0.353274	0.029067	0.1	0.593499	0.544554	1	1	1
M851T310	2.024148	0.007111	0.1	0.593499	0.544554	1	1	1
M854T349	0.433947	0.038949	0.1	0.593499	0.544554	1	1	1
M856T369	0.490814	0.048497	0.1	0.593499	0.544554	1	1	1
M858T267	2.513727	0.043072	0.1	0.593499	0.544554	1	1	1
M861T277	45.93709	0.024863	0.1	0.593499	0.544554	1	1	1
M864T474	3.188264	0.047067	0.1	0.593499	0.544554	1	1	1
M865T467	2.956862	0.031866	0.1	0.593499	0.544554	1	1	1
M871T483	2.925601	0.033392	0.1	0.593499	0.544554	1	1	1
M877T344	0.343559	0.030063	0.1	0.593499	0.544554	1	1	1
M877T479	2.386593	0.039474	0.1	0.593499	0.544554	1	1	1
M877T525	1.542309	0.008665	0.1	0.593499	0.544554	1	1	1
M878T295	2.3844	0.020574	0.1	0.593499	0.544554	1	1	1

M878T344	0.330228	0.018991	0.1	0.593499	0.544554	1	1	1
M880T478	2.225292	0.034907	0.1	0.593499	0.544554	1	1	1
M884T280	43.53897	0.026868	0.1	0.593499	0.544554	1	1	1
M887T302	1.622523	0.038959	0.1	0.593499	0.544554	1	1	1
M890T256	0.529569	0.009234	0.1	0.593499	0.544554	1	1	1
M894T459	2.02615	0.0201	0.1	0.593499	0.544554	1	1	1
M896T282	12.18692	0.039688	0.1	0.593499	0.544554	1	1	1
M897T310	2.611834	0.045082	0.1	0.593499	0.544554	1	1	1
M897T469	1.575582	0.046329	0.1	0.593499	0.544554	1	1	1
M900T344	0.413407	0.049532	0.1	0.593499	0.544554	1	1	1
M901T336	0.333063	0.044322	0.1	0.593499	0.544554	1	1	1
M902T248	7.703587	0.009464	0.1	0.593499	0.544554	1	1	1
M905T322	3.143854	0.049951	0.1	0.593499	0.544554	1	1	1
M908T283	11.62555	0.012389	0.1	0.593499	0.544554	1	1	1
M915T370	2.226187	0.03269	0.1	0.593499	0.544554	1	1	1
M919T284	42.69699	0.042218	0.1	0.593499	0.544554	1	1	1
M919T285	41.84278	0.044096	0.1	0.593499	0.544554	1	1	1
M922T424	2.052355	0.048158	0.1	0.593499	0.544554	1	1	1
M923T229	9.608232	0.023472	0.1	0.593499	0.544554	1	1	1
M930T309	3.058545	0.035173	0.1	0.593499	0.544554	1	1	1
M941T260	19.50602	0.018965	0.1	0.593499	0.544554	1	1	1
M944T309	1.745405	0.028649	0.1	0.593499	0.544554	1	1	1
M951T344	0.215721	0.002691	0.1	0.593499	0.544554	1	1	1
M954T317	0.348762	0.0255	0.1	0.593499	0.544554	1	1	1
M955T337	0.502084	0.02761	0.1	0.593499	0.544554	1	1	1
M956T337	0.586692	0.010446	0.1	0.593499	0.544554	1	1	1
M967T224	21.93492	0.038982	0.1	0.593499	0.544554	1	1	1
M970T301	0.600506	0.000292	0.1	0.593499	0.544554	1	1	1
M973T337	0.588214	0.042368	0.1	0.593499	0.544554	1	1	1
M980T228	540.9661	0.03453	0.1	0.593499	0.544554	1	1	1
M981T228	162.3644	0.036574	0.1	0.593499	0.544554	1	1	1
M990T259	0.459981	0.012108	0.1	0.593499	0.544554	1	1	1
M993T317	0.427343	0.005926	0.1	0.593499	0.544554	1	1	1
M994T296	0.328953	0.02434	0.1	0.593499	0.544554	1	1	1
M995T337	0.457312	0.034403	0.1	0.593499	0.544554	1	1	1
M996T338	0.265662	0.012885	0.1	0.593499	0.544554	1	1	1

sample	rawCV	cv	regulated	QC_1	QC_2	QC_3	QC_4	QC_5
VPA:Contr	0.07878	0.077657	up	4304.935	3475.001	3705.209	3408.468	3737.968
VPA:Contr	0.085429	0.068787	down	2412.405	2443.82	2615.004	2715.148	2177.63
VPA:Contr	0.104361	0.09291	up	1829.016	2127.766	1966.272	1554.658	1966.809
VPA:Contr	0.603635	0.285894	down	1253.232	844.248	1960.113	1034.68	1109.963
VPA:Contr	0.232292	1.91E-11	down	3446.332	3446.332	3446.332	3446.332	3446.332
VPA:Contr	0.196863	0.181803	up	1722.296	1865.354	2555.229	1477.963	2135.656
VPA:Contr	0.125563	0.093223	up	1583.906	1940.225	1679.179	1888.215	1612.56
VPA:Contr	0.216712	1.92E-12	down	1878.168	1878.168	1878.168	1878.168	1878.168
VPA:Contr	0.286377	0.199894	down	1231.444	1317.676	1584.598	791.278	1482.014
VPA:Contr	0.140131	0.134623	up	1115.741	934.7094	1333.133	1000.068	1156.122
VPA:Contr	0.185805	0.158052	down	2299.222	1882.793	1679.282	1665.253	1493.34
VPA:Contr	0.168524	0.087724	up	2266.137	2511.447	1853.241	2331.24	2267.415
VPA:Contr	0.24702	0.2289	up	1708.723	2634.143	2441.072	1315.047	2039.178
VPA:Contr	0.104495	0.048181	up	7611.696	7420.455	8432.788	7254.645	7655.056
VPA:Contr	0.187978	0.145204	up	1716.452	1213.898	1511.836	1115.328	1539.794
VPA:Contr	0.26113	0.096188	down	676.1948	713.9579	781.1004	572.7838	760.081
VPA:Contr	0.274996	0.145424	down	2790.585	2880.396	3824.75	2383.828	3089.463
VPA:Contr	0.176707	9.92E-12	down	2477.076	2477.076	2477.076	2477.076	2477.076
VPA:Contr	0.134109	0.133815	down	2412.898	1699.054	1690.975	1908.973	1707.516
VPA:Contr	0.266236	7.55E-12	down	2453.364	2453.364	2453.364	2453.364	2453.364
VPA:Contr	0.329226	0.227018	down	1071.671	1064.077	1078.914	823.3916	1273.42
VPA:Contr	0.318712	1.22E-12	up	10129.77	10129.77	10129.77	10129.77	10129.77
VPA:Contr	0.196124	0.193735	down	4040.024	2725.799	4494.375	2853.62	3691.705
VPA:Contr	0.356804	0.296016	down	778.6411	1171.711	700.48	517.2791	1103.473
VPA:Contr	0.07534	0.065168	down	57828.71	47054.52	52712.77	55058.97	50767.94
VPA:Contr	0.050061	0.039367	down	50834.52	48082.03	49242.84	47035.77	45235.36
VPA:Contr	0.101112	0.100909	down	38688.36	33649.52	28503.28	31542.7	37487.93
VPA:Contr	0.395247	0.274504	down	10559.53	7226.541	11352	16656.17	8914.781
VPA:Contr	0.227687	0.140534	down	6053.472	6498.757	7278.69	5673.308	8506.323
VPA:Contr	0.141793	0.129362	down	5269.34	4105.177	4643.016	5309.253	4224.375
VPA:Contr	0.136876	0.136881	down	7005.249	4551.425	5926.685	5995.11	6809.462
VPA:Contr	0.126358	0.083306	down	9683.517	7967.474	8063.639	8855.137	8854.321
VPA:Contr	0.104548	0.07732	down	24133.47	21685.46	18944.3	23771.83	21487.47
VPA:Contr	0.055719	0.054598	down	21846.78	19636.24	20447.66	22360.23	19176.4
VPA:Contr	0.077883	0.036176	down	15371.8	15635.54	14220.47	16039.57	15223.37
VPA:Contr	0.086486	0.083416	down	26140.88	27403.55	29838.47	30714.76	24057.7
VPA:Contr	0.140829	0.129311	down	6534.115	7841.483	6511.157	6394.151	5716.177
VPA:Contr	0.090146	0.087777	down	4474.302	4999.835	3725.163	4754.574	4534.113
VPA:Contr	0.389418	3.10E-12	down	1866.596	1866.596	1866.596	1866.596	1866.596
VPA:Contr	0.158413	0.131008	down	2423.555	1693.596	2491.776	2581.511	2168.6
VPA:Contr	0.327498	2.58E-09	up	7124.84	7124.84	7124.84	7124.84	7124.84
VPA:Contr	0.320978	0.198815	down	16506.89	19780.15	15108.88	15724.04	25635.69
VPA:Contr	0.296117	0.222005	down	11184.12	19939.89	11886.64	12793.84	11925.58
VPA:Contr	0.093442	0.053129	up	1078.503	1076.353	1193.689	1013.038	1121.069
VPA:Contr	0.126708	0.101867	down	8720.661	9485.53	8812.862	11583.23	9619.661
VPA:Contr	0.068136	0.061259	down	5653.084	5427.553	4724.854	5750.574	5487.605
VPA:Contr	0.293815	1.74E-13	up	74639.74	74639.74	74639.74	74639.74	74639.74
VPA:Contr	0.183637	0.088962	up	1191.411	1401.257	1224.045	1030.848	1256.603
VPA:Contr	0.054797	0.053776	down	8028.595	8223.557	7930.415	8857.015	7384.028
VPA:Contr	0.093223	0.066242	down	4065.73	3726.3	3738.073	4429.262	3696.819
VPA:Contr	0.741938	3.10E-09	up	1910.078	1910.078	1910.078	1910.078	1910.078
VPA:Contr	0.161056	0.152682	down	6700.414	5141.508	5354.387	7601.395	5163.117
VPA:Contr	0.692665	2.76E-11	up	13611.7	13611.7	13611.7	13611.7	13611.7
VPA:Contr	0.227234	5.01E-12	down	3716.689	3716.689	3716.689	3716.689	3716.689
VPA:Contr	0.191547	0.197065	down	1172.446	1079.101	1250.944	1279.282	1471.952
VPA:Contr	0.429105	1.66E-11	up	1497.355	1497.355	1497.355	1497.355	1497.355
VPA:Contr	0.499735	0.211255	up	775.8554	871.5176	644.8437	446.1957	908.635

VPA:Contr	0.050659	0.050638	down	251497.2	255713.4	242197.7	272727.9	255322.8
VPA:Contr	0.064573	3.03E-12	down	81900.3	81900.3	81900.3	81900.3	81900.3
VPA:Contr	0.136074	0.070428	down	3575.976	3454.151	4134.422	3310.984	3642.773
VPA:Contr	0.211302	5.32E-12	down	1306.497	1306.497	1306.497	1306.497	1306.497
VPA:Contr	0.227071	0.162517	down	3108.187	3023.441	3304.657	4590.446	3095.082
VPA:Contr	0.182035	0.172193	up	4673.403	3961.415	6216.213	4231.598	4516.112
VPA:Contr	0.138721	0.118991	down	1469.018	1500.347	1281.464	1103.709	1620.182
VPA:Contr	0.250384	2.49E-12	up	1467.076	1467.076	1467.076	1467.076	1467.076
VPA:Contr	0.157823	0.11481	down	3396.755	2772.784	2631.79	3126.156	2444.932
VPA:Contr	0.279237	0.182154	down	1845.982	1785.944	1214.837	1413.4	2064.05
VPA:Contr	0.10937	0.046249	up	9356.19	9751.521	9242.432	9715.286	10586.53
VPA:Contr	0.105894	0.014176	up	4931.304	5001.084	4789.603	4997.218	4935.718
VPA:Contr	0.117793	0.11733	up	2271.083	2583.93	1756.873	2013.436	2224.092
VPA:Contr	0.070522	0.051155	down	18538.58	16165.09	18005.35	18509.26	17527.95
VPA:Contr	0.107737	0.104078	down	6418.008	6275.913	5643.166	6546.212	6056.266
VPA:Contr	0.152863	0.140885	down	1689.547	1895.684	1370.925	1272.623	1835.721
VPA:Contr	0.28881	3.63E-11	up	2809.487	2809.487	2809.487	2809.487	2809.487
VPA:Contr	0.153747	0.146205	down	8930.923	5709.601	8034.841	7979.117	7166.5
VPA:Contr	0.235303	0.13035	up	1279.044	1625.01	1215.668	1346.134	1414.042
VPA:Contr	0.151027	5.05E-12	down	2952.555	2952.555	2952.555	2952.555	2952.555
VPA:Contr	0.100115	0.07943	up	1550.331	1384.105	1229.113	1351.512	1543.873
VPA:Contr	0.297743	1.31E-11	up	3672.675	3672.675	3672.675	3672.675	3672.675
VPA:Contr	0.317717	0.028518	up	3262.406	3196.946	3190.253	3428.958	3135.959
VPA:Contr	0.129133	0.06214	down	1834.393	1842.788	2015.329	2074.677	2117.027
VPA:Contr	0.202432	0.114002	down	1419.953	1316.282	1482.842	1216.372	1735.699
VPA:Contr	0.155444	0.115853	up	2334.361	2117.205	2191.176	2159.859	2898.797
VPA:Contr	0.060853	0.054014	down	15780.03	14707.49	16089.57	14746.04	17140.87
VPA:Contr	0.223595	1.32E-11	up	7898.995	7898.995	7898.995	7898.995	7898.995
VPA:Contr	0.117932	0.117473	down	1013.654	909.9027	907.4904	1170.359	815.5678
VPA:Contr	0.272048	0.208746	down	13843.49	12448.45	12910.74	8054.924	15924.1
VPA:Contr	0.346385	0.02856	up	1704.852	1796.932	1634.935	1753.923	1723.515
VPA:Contr	0.058797	0.056623	down	29292.66	28954.35	28336.84	33228.57	28556.14
VPA:Contr	0.076759	0.074114	down	9253.168	8429.762	10050.58	10638.01	9368.747
VPA:Contr	0.080577	0.062137	up	2835.712	2470.694	2578.999	2499.333	2855.616
VPA:Contr	0.215141	0.179367	down	1232.274	1097.389	1785.106	1502.359	1118.529
VPA:Contr	0.112931	0.06762	up	5410.327	5058.952	4901.499	5751.279	4741.168
VPA:Contr	0.087164	0.021009	up	19880.1	20842.35	19425.8	20218.85	20116.89
VPA:Contr	0.141737	3.00E-12	down	8074.145	8074.145	8074.145	8074.145	8074.145
VPA:Contr	0.118966	0.060694	down	2622.033	2501.571	2296.573	2538.962	2813.403
VPA:Contr	0.066609	0.061565	down	16514.21	15647.37	18991.98	17037.94	17512.65
VPA:Contr	0.035984	0.022605	down	31111.45	30867.09	31280.98	32385.24	30026.29
VPA:Contr	0.118126	0.091399	down	10927.64	11933.3	10699.09	12912.18	9857.357
VPA:Contr	0.23562	0.232964	down	1852.685	1155.829	1546.635	1431.246	1034.561
VPA:Contr	0.237412	2.48E-11	up	23024.07	23024.07	23024.07	23024.07	23024.07
VPA:Contr	0.185731	0.171082	down	3047.887	4623.087	3401.403	3550.604	3048.119
VPA:Contr	0.298662	1.87E-11	down	1371.9	1371.9	1371.9	1371.9	1371.9
VPA:Contr	0.030089	0.030042	down	1325401	1248102	1331478	1323478	1275682
VPA:Contr	0.038703	0.022257	down	342997.2	341605.3	330564.5	356693.5	340927
VPA:Contr	0.149454	4.29E-12	up	12707.82	12707.82	12707.82	12707.82	12707.82
VPA:Contr	0.251312	1.25E-12	up	26270.03	26270.03	26270.03	26270.03	26270.03
VPA:Contr	0.067815	1.47E-12	down	70952.63	70952.63	70952.63	70952.63	70952.63
VPA:Contr	0.083839	0.076183	down	8882.308	8144.615	7676.658	7721.979	7195.499
VPA:Contr	0.120117	0.092325	down	10970.38	13162.66	11264.07	9676.084	10936.57
VPA:Contr	0.067984	0.053712	down	6115.346	5965.012	5659.099	6249.318	5308.939
VPA:Contr	0.208329	0.113304	down	6398.647	6013.303	7373.326	5116.524	7012.126
VPA:Contr	0.258977	0.186309	up	2611.396	3355.347	2688.485	1704.95	2940.695
VPA:Contr	0.224972	0.213597	down	1360.056	1320.696	1985.253	1310.821	980.4228
VPA:Contr	0.381272	3.14E-12	up	4084.724	4084.724	4084.724	4084.724	4084.724

VPA:Contr	0.441064	3.46E-08	down	1408.634	1408.634	1408.634	1408.634	1408.634
VPA:Contr	0.283921	0.017019	down	1519.164	1541.488	1586.851	1501.481	1540.173
VPA:Contr	0.209289	9.01E-12	down	1682.992	1682.992	1682.992	1682.992	1682.992
VPA:Contr	0.198971	4.31E-12	up	883.2689	883.2689	883.2689	883.2689	883.2689
VPA:Contr	0.396186	4.38E-11	down	2343.112	2343.112	2343.112	2343.112	2343.112
VPA:Contr	0.231032	3.94E-12	up	4996.521	4996.521	4996.521	4996.521	4996.521
VPA:Contr	0.135146	0.073786	up	758.4641	967.7146	845.4132	857.7632	823.9595
VPA:Contr	0.254833	0.251478	up	2587.021	1651.674	2430.685	2985.793	1359.989
VPA:Contr	0.036501	0.021298	down	147972.7	143757	146178.8	152198.8	142586.2
VPA:Contr	0.031647	0.017795	down	45455.59	43734.93	44292.32	45666.6	44315.7
VPA:Contr	0.032677	0.02626	down	204886.8	198457.9	214984.7	199788.1	202443.5
VPA:Contr	0.058446	0.052221	down	63759.34	58699.64	66939.84	57421.56	63309.46
VPA:Contr	0.158299	0.076362	down	1359.315	1225.318	1507.04	1511.891	1294.443
VPA:Contr	0.07984	1.93E-12	up	11746.32	11746.32	11746.32	11746.32	11746.32
VPA:Contr	0.184351	0.161203	down	1522.568	938.2006	1138.99	1001.412	1234.902
VPA:Contr	0.218798	4.49E-12	up	2135.228	2135.228	2135.228	2135.228	2135.228
VPA:Contr	0.191895	0.192957	down	3853.58	5179.294	5228.162	6248.606	3560.771
VPA:Contr	0.183334	0.182959	down	5020.273	5233.426	5989.633	6709.584	3613.45
VPA:Contr	0.250554	0.17864	down	2418.585	1852.451	3123.354	1952.89	2238.419
VPA:Contr	0.147505	2.30E-12	down	22241.8	22241.8	22241.8	22241.8	22241.8
VPA:Contr	0.112408	0.006677	down	1755.248	1736.196	1775.298	1747.474	1750.458
VPA:Contr	0.214291	0.18591	up	2171.281	2343.146	1841.929	2103.499	3002.495
VPA:Contr	0.101327	0.063853	up	1305.716	1213.388	1336.618	1167.672	1419.907
VPA:Contr	0.059715	0.037596	up	33610.48	31288.24	30829.41	34353.69	32692.21
VPA:Contr	0.266131	0.203607	down	12764.19	10515.08	11368.77	9613.224	17253.34
VPA:Contr	0.051416	1.20E-12	down	24844.53	24844.53	24844.53	24844.53	24844.53
VPA:Contr	0.101434	0.090434	down	9571.398	7840.955	7771.903	8485.449	8324.576
VPA:Contr	0.196093	0.185456	down	3052.298	4014.214	4675.552	3037.614	3829.472
VPA:Contr	0.048364	6.73E-10	down	40343.27	40343.27	40343.27	40343.27	40343.27
VPA:Contr	0.039699	0.037192	down	14426.86	13248.93	14190.66	13184.05	13190.02
VPA:Contr	0.170076	0.124413	up	4740.769	5000.955	5248.037	3979.97	5332.458
VPA:Contr	0.0998	0.068976	down	19587.79	22126.82	17921.52	18453.22	20380.24
VPA:Contr	0.117201	0.115956	down	7197.745	8259.416	6026.079	7259.043	8737.022
VPA:Contr	0.065567	9.96E-13	up	27788.68	27788.68	27788.68	27788.68	27788.68
VPA:Contr	0.291328	5.87E-14	up	4484.785	4484.785	4484.785	4484.785	4484.785
VPA:Contr	0.19221	1.73E-09	up	2414.929	2414.929	2414.929	2414.929	2414.929
VPA:Contr	0.13151	0.120578	down	9070.722	8356.854	7960.665	8924.904	6533.535
VPA:Contr	0.076689	0.060469	down	3170.825	2842.22	3214.792	2929.031	2738.216
VPA:Contr	0.163058	0.163099	up	3393.958	2257.951	2409.169	2686.323	3086.748
VPA:Contr	0.102001	0.071221	down	3202.277	3221.721	3841.109	3371.839	3115.787
VPA:Contr	0.293731	0.269675	up	4002.824	4144.702	1521.324	3249.328	3061.919
VPA:Contr	0.390429	1.15E-11	down	10192.06	10192.06	10192.06	10192.06	10192.06
VPA:Contr	0.161634	0.15812	down	1702.598	2435.227	1863.214	1465.07	1904.635
VPA:Contr	0.048643	0.048389	up	55602.9	48612.2	54022.82	55300.26	50951.87
VPA:Contr	0.066114	4.33E-12	up	23865.58	23865.58	23865.58	23865.58	23865.58
VPA:Contr	0.127196	0.107543	down	1113.048	803.6622	887.2097	903.3983	845.2546
VPA:Contr	0.099871	0.056269	up	4840.97	4182.337	4452.598	4501.145	4947.299
VPA:Contr	0.117472	1.42E-11	up	6076.996	6076.996	6076.996	6076.996	6076.996
VPA:Contr	0.136422	0.039307	down	6329.154	6680.861	6091.63	6830.742	6241.274
VPA:Contr	0.115959	0.055992	down	3924.405	4276.172	3988.236	4636.284	4153.657
VPA:Contr	0.187271	0.173731	down	1436.612	1107.908	1592.537	1661.956	1202.627
VPA:Contr	0.167835	0.09454	down	8251.105	9223.179	8562.366	7117.327	9697.401
VPA:Contr	0.104599	0.101558	down	13972.15	10771.27	12879.99	12458.23	11291.12
VPA:Contr	0.159342	0.053539	down	1631.963	1524.392	1460.587	1722.633	1531.475
VPA:Contr	0.054336	0.044913	up	42974.96	38724.08	41503.44	38117.24	42564.52
VPA:Contr	0.135028	0.132337	up	1220.295	1461.54	1142.177	1309.305	1179.888
VPA:Contr	0.111186	0.059034	down	18141.37	18964.83	16195.88	17069.1	19061.03
VPA:Contr	0.238748	2.14E-11	down	1868.968	1868.968	1868.968	1868.968	1868.968

VPA:Contr	0.199837	0.177568	down	2374.331	1503.223	2273.77	1634.101	1615.365
VPA:Contr	0.096621	0.081273	up	4180.244	4174.904	4799.201	3646.106	4124.058
VPA:Contr	0.292337	0.270896	up	863.8142	742.4552	1490.891	1301.603	1108.146
VPA:Contr	0.100506	1.07E-13	up	18136.02	18136.02	18136.02	18136.02	18136.02
VPA:Contr	0.058647	0.045469	up	13172.03	13720.41	14411.74	13976.45	15078.61
VPA:Contr	0.207733	0.204455	up	10965.52	9304.775	15698.18	9579.214	11351.59
VPA:Contr	0.135367	0.132911	up	829.1936	1118.354	802.2537	882.6926	1025.68
VPA:Contr	0.518745	0.115829	up	9066.705	10128.77	7843.006	11194.03	8541.949
VPA:Contr	0.113264	0.108712	down	2525.049	2633.712	3163.589	2780.726	2215.288
VPA:Contr	0.355616	3.59E-11	up	1038.682	1038.682	1038.682	1038.682	1038.682
VPA:Contr	0.434592	1.90E-11	up	14386.65	14386.65	14386.65	14386.65	14386.65
VPA:Contr	0.190826	0.17428	down	5700.145	7219.955	4611.673	4706.776	4866.556
VPA:Contr	0.174915	0.086683	up	1415.053	1203.737	1179.859	1506.082	1285.912
VPA:Contr	0.195005	0.170103	down	1154.8	1719.92	1902.757	1466.813	1874.495
VPA:Contr	0.130264	0.10826	down	13017.24	10046.94	11399.97	12156.94	9601.04
VPA:Contr	0.188826	0.13521	down	816.3884	653.9883	737.0744	870.9826	571.3377
VPA:Contr	0.128555	0.039302	up	2803.761	2579.224	2937.43	2697.777	2739.13
VPA:Contr	0.08224	0.081819	down	11419.17	11477.22	11111.73	11957.81	9863.471
VPA:Contr	0.228559	0.084835	up	16734.98	14517.35	19016.78	15400.07	16219.46
VPA:Contr	0.099319	0.0964	up	7863.981	10147.12	9571.04	7804.638	9266.268
VPA:Contr	0.107826	0.049222	down	11185.08	11948.07	10467.12	12164.49	11152.83
VPA:Contr	0.206264	0.206834	down	6622.701	4015.943	7070.746	4884.589	5292.56
VPA:Contr	0.316508	1.31E-11	down	2707.335	2707.335	2707.335	2707.335	2707.335
VPA:Contr	0.19847	0.188559	up	2227.892	3304.614	3529.294	3669.54	3966.256
VPA:Contr	0.215561	2.72E-10	down	2160.84	2160.84	2160.84	2160.84	2160.84
VPA:Contr	0.122656	0.079594	down	4587.017	5178.651	4298.036	5366.601	4473.374
VPA:Contr	0.17619	0.132136	down	7300.962	10549.51	8962.034	7220.391	8917.252
VPA:Contr	0.054287	0.038561	down	18985.12	17884.99	20157.05	18178.88	18583.19
VPA:Contr	0.135831	0.135854	up	3278.628	3478.777	3219.571	4136.651	2868.686
VPA:Contr	0.086181	0.081342	down	10033.25	9708.999	10737.51	9156.311	11226.76
VPA:Contr	0.089636	0.088838	down	16363.3	14345.54	15274.12	18374.56	15114.26
VPA:Contr	0.040062	0.002785	down	51755.88	52036.38	51609.7	51976.84	51746.84
VPA:Contr	0.131485	0.049539	up	12429.04	12811.89	14151.98	13268.41	12273.78
VPA:Contr	0.205331	0.12322	down	150893.7	129701	193700.8	163085.3	156183.1
VPA:Contr	0.167368	0.152005	up	2583.612	3960.903	2600.027	3353.635	3106.211
VPA:Contr	0.069687	4.18E-12	down	25712.32	25712.32	25712.32	25712.32	25712.32
VPA:Contr	0.131539	0.088517	up	6348.611	8139.405	6637.706	6470.395	7242.316
VPA:Contr	0.209656	0.123364	down	72381.86	61265.39	83598.6	84242.35	86365.03
VPA:Contr	0.173588	0.027433	down	2237.343	2336.479	2134.295	2291.819	2262.375
VPA:Contr	0.19692	0.099717	down	2026.52	1494.413	1976.053	1672.518	1795.218
VPA:Contr	0.040895	5.58E-13	down	167451.8	167451.8	167451.8	167451.8	167451.8
VPA:Contr	0.044104	0.028045	down	71646.54	71474.31	76443.56	69970.96	72335.41
VPA:Contr	0.083814	0.018937	down	20861.21	20023.36	20457.45	20022.31	21059.61
VPA:Contr	0.193981	0.194382	up	8949.742	9990.449	6195.584	6764.137	10481.36
VPA:Contr	0.129985	0.130985	down	23570.19	21975.07	22175.69	20344.49	18506.22
VPA:Contr	0.129677	0.119556	down	11227.42	8687.554	9840.849	8634.202	9901.693
VPA:Contr	0.193346	0.187532	down	2548.603	2889.405	3050.028	2190.09	2964.895
VPA:Contr	0.045547	0.039024	down	352696	329688.3	376849.8	348984.4	351278.5
VPA:Contr	0.175814	9.87E-13	down	6920.734	6920.734	6920.734	6920.734	6920.734
VPA:Contr	0.047022	0.046319	down	166405.5	146672.9	162171.2	167542	158002.9
VPA:Contr	0.079596	0.063579	down	44400.28	42593.8	44827.03	39101.46	48183.5
VPA:Contr	0.043299	0.01871	down	274730	285912.2	276631.4	268523.2	279012.1
VPA:Contr	0.113547	1.13E-12	up	1302.811	1302.811	1302.811	1302.811	1302.811
VPA:Contr	0.093293	0.064272	up	2560.108	2329.835	2302.689	2500.143	2105.153
VPA:Contr	0.182234	2.08E-12	up	3082.531	3082.531	3082.531	3082.531	3082.531
VPA:Contr	0.187505	0.105776	up	763.1598	927.0998	939.8796	710.8127	937.4014
VPA:Contr	0.330458	3.85E-12	up	2702.238	2702.238	2702.238	2702.238	2702.238
VPA:Contr	0.150938	0.140728	up	21786.5	14049.69	17590.58	16047.9	19241.62

VPA:Contr	0.023158	0.008748	down	919887.9	922386.4	903242.8	929892.9	915850.5
VPA:Contr	0.118495	0.105064	down	45883.62	40930.19	39947.36	43053.65	41517.44
VPA:Contr	0.032786	1.71E-10	down	392194.8	392194.8	392194.8	392194.8	392194.8
VPA:Contr	0.066814	0.04585	up	4359.805	3844.327	4293.199	4456.857	4303.678
VPA:Contr	0.192885	0.121153	up	1230.87	988.769	1231.891	983.7083	1183.772
VPA:Contr	0.035938	0.032134	down	201351.1	202513.1	207225.7	188015.7	202790.2
VPA:Contr	0.032875	0.019431	down	95259.42	94784.69	98864.95	92544.5	95654.12
VPA:Contr	0.018262	0.011974	down	327498.9	328600.4	323270.6	335816.9	331504.6
VPA:Contr	0.029191	0.009049	down	145110.1	146711.3	145056.1	147521.1	144253
VPA:Contr	0.120784	0.058881	down	41339.01	42107.04	43063.21	40190.26	42699.15
VPA:Contr	0.261895	0.184019	down	9332.28	10636.24	11980.55	6424.674	10968.64
VPA:Contr	0.147387	0.133373	up	3191.546	3807.869	3465.922	3302.916	4205.107
VPA:Contr	0.168418	0.119202	down	11031.17	9987.976	13310.56	12149.72	9922.809
VPA:Contr	0.100493	0.007774	down	9204.912	9160.292	9348.465	9114.892	9216.488
VPA:Contr	0.134167	0.134312	up	6324.902	6515.907	5098.833	5430.274	7577.312
VPA:Contr	0.074363	0.028716	down	25481.45	25322.81	24859.17	26943.42	24669.9
VPA:Contr	0.379522	0.241657	up	3377.759	1737.378	3055.826	3887.223	2562
VPA:Contr	0.193137	0.050816	down	13951.75	12415.01	14417.48	14166.89	13014.23
VPA:Contr	0.125835	0.1262	down	38664.44	49963.59	43709.86	33290.13	40096.97
VPA:Contr	0.110155	0.039689	up	2079.082	2265.204	2066.62	2008.236	2045.739
VPA:Contr	0.164573	0.151434	up	4315.695	5889.829	4102.778	5938.175	4495.826
VPA:Contr	0.020097	0.020097	down	954671.9	911382.4	928774.5	940622.3	927929.9
VPA:Contr	0.032045	0.030545	down	420671	443009.7	423351.5	440915.8	455294.1
VPA:Contr	0.163173	0.138674	up	6054.59	8504.238	6792.641	6042.262	6826.681
VPA:Contr	0.067042	0.064134	down	30317.82	34453.26	35825.85	30067.46	32747.1
VPA:Contr	0.25058	0.252133	down	25494.72	19641.74	16085.02	16719.65	16704.87
VPA:Contr	0.103217	9.01E-12	down	74160.17	74160.17	74160.17	74160.17	74160.17
VPA:Contr	0.014958	0.014529	down	666306.9	642311	660080.2	650762.5	651058.9
VPA:Contr	0.095196	0.014113	down	199848.3	195602.6	194527.2	202828.3	196589.4
VPA:Contr	0.030557	0.028433	down	313479.9	306656.7	292571.8	320976.6	311031.2
VPA:Contr	0.042586	0.023551	down	85064.49	92242.98	88322.08	88622.66	88627.27
VPA:Contr	0.230114	0.229654	down	28723.09	24130.13	29030.6	40158.09	21867.71
VPA:Contr	0.100061	0.093512	down	26183.32	28634.67	22609.15	26023.85	28892.47
VPA:Contr	0.109647	0.059926	down	14158.71	12935.98	12507.81	14680.32	12604.01
VPA:Contr	0.078476	0.035853	down	9825.762	9878.858	10360.66	9191.191	10070.31
VPA:Contr	0.087928	0.065702	down	4449.028	4264.077	3980.884	4880.659	4664.605
VPA:Contr	0.308011	0.293727	up	1102.725	2193.35	1625.53	1011.656	2151.654
VPA:Contr	0.087403	0.068994	down	2727.587	2484.549	2936.315	2980.034	2864.335
VPA:Contr	0.312913	0.291049	down	4274.472	3857.759	4876.337	7949.379	4008.985
VPA:Contr	0.210299	0.166115	down	8676.153	12981.82	9799.665	9545.273	10975.22
VPA:Contr	0.160191	0.089035	down	9718.686	11270.52	9679.29	9135.079	8528.377
VPA:Contr	0.168743	0.161349	up	3601.145	5386.031	4243.949	5521.82	5554.991
VPA:Contr	0.113325	5.70E-12	down	4408.577	4408.577	4408.577	4408.577	4408.577
VPA:Contr	0.10868	0.107844	down	18517.23	20216.29	24598.84	18011.67	19231.3
VPA:Contr	0.0291	0.008885	down	371325.8	369658.8	375801.2	365269.9	373753.6
VPA:Contr	0.105679	0.105499	down	16473.43	13689.74	15699.89	18005.7	14116.81
VPA:Contr	0.123212	0.122041	up	1461.808	1222.769	1495.597	1429.53	1501.719
VPA:Contr	0.040307	2.52E-10	down	184070.9	184070.9	184070.9	184070.9	184070.9
VPA:Contr	0.086032	0.084593	down	8928.846	11420.3	10048.61	9819.609	10608.71
VPA:Contr	0.062531	7.44E-13	down	22231.2	22231.2	22231.2	22231.2	22231.2
VPA:Contr	0.165836	0.158061	down	11724.7	8875.601	8431.002	8998.264	7293.591
VPA:Contr	0.200907	5.41E-12	up	10317.22	10317.22	10317.22	10317.22	10317.22
VPA:Contr	0.079886	0.065525	down	72564.73	66618.34	69135.89	79143.09	65017.04
VPA:Contr	0.053711	0.050136	down	202881.6	194276.3	221284.6	213986.6	192461.5
VPA:Contr	0.093841	0.054099	up	7433.757	8127.663	8179.311	7495.629	8225.732
VPA:Contr	0.059312	0.041561	down	106470.9	101754.6	115149.8	107219.6	102049.3
VPA:Contr	0.04107	0.036084	down	9714.668	10244.98	10613.77	10083.48	10811.04
VPA:Contr	0.156172	0.132344	down	38828.37	40877.85	51421.87	34567.95	47599.13

VPA:Contr	0.351387	0.191787	down	9626.351	7081.215	12912.28	8550.149	8624.318
VPA:Contr	0.059345	0.046777	down	13038.34	12135.51	13714.73	13607.03	13864.76
VPA:Contr	0.130775	7.47E-12	down	10468.34	10468.34	10468.34	10468.34	10468.34
VPA:Contr	0.232572	0.172582	down	78634.46	66670.37	85360.95	69392.13	47607.2
VPA:Contr	0.08021	1.01E-12	down	4417214	4417214	4417214	4417214	4417214
VPA:Contr	0.13301	0.119656	down	1915392	1830858	1766938	2459747	1824014
VPA:Contr	0.105312	0.089734	down	5587837	5817656	5633947	7109751	5706420
VPA:Contr	0.090119	0.077401	down	2334164	2727601	2404875	2833110	2807380
VPA:Contr	0.081464	0.081532	down	973143	821680.8	1004339	845911.3	840665.2
VPA:Contr	0.048773	2.80E-12	down	56148.32	56148.32	56148.32	56148.32	56148.32
VPA:Contr	0.074321	0.074167	down	484481.7	427017.9	491296.9	410892.4	419172.8
VPA:Contr	0.123606	0.042529	down	25794.63	27785.6	24065.18	26328.05	26617.97
VPA:Contr	0.073419	0.070132	down	154069.4	132370.3	140541	129884.2	125802.7
VPA:Contr	0.038474	1.95E-09	down	1684768	1684768	1684768	1684768	1684768
VPA:Contr	0.141979	0.028307	down	19739.33	19037.98	18608.01	20321.82	19107.4
VPA:Contr	0.045156	0.031271	down	815929.8	818507	782884.4	856663.1	795458.9
VPA:Contr	0.043101	0.038019	down	237885.7	238562.5	228679.9	232305.8	227675.3
VPA:Contr	0.04513	0.040807	down	105890.6	93715.19	100736.3	100749.7	100345.6
VPA:Contr	0.071148	0.057102	down	25235.08	25502.11	29195.93	24493.76	26368.87
VPA:Contr	0.065021	0.060023	down	22264.82	23839.55	23390.74	20076.59	22136.01
VPA:Contr	0.159588	0.158739	down	7560.58	7903.226	7189.129	7707.56	10937.61
VPA:Contr	0.043552	0.037854	down	57212.25	53005.71	57072.64	51844.65	53457.24
VPA:Contr	0.15818	0.143227	down	33723.07	30780.01	38174.58	46414.24	32818.51
VPA:Contr	0.185666	0.171478	up	948.3768	877.8503	1367.392	843.8499	1076.26
VPA:Contr	0.266966	0.222509	down	45704.88	78002.32	46039.81	47664.61	44803.58
VPA:Contr	0.067052	0.047131	down	5178.248	4559.304	4689.243	4683.668	4954.095
VPA:Contr	0.253485	0.222127	down	102875.7	106423.2	115248.4	98164.51	173058.4
VPA:Contr	0.204488	0.188836	down	54176.48	57545.55	59637.19	88387.57	56299
VPA:Contr	0.040263	0.03897	down	37211.16	37778.76	39652.22	35299.44	36828.72
VPA:Contr	0.119713	0.10434	down	17314.54	12818.15	17374.69	14555.89	14805.33
VPA:Contr	0.099977	0.099215	down	15151.21	15115.67	18133.51	13216.16	15998.31
VPA:Contr	0.086529	0.046861	down	8728.947	8459.565	9335.135	8941.398	8120.093
VPA:Contr	0.157445	0.036326	down	18752.99	17914.48	20124.82	18970.84	18319.87
VPA:Contr	0.034218	0.007275	down	76255.11	75550.3	77305.37	75762.5	76126.28
VPA:Contr	0.138414	0.110423	down	55659.28	38338.23	48996.9	50526.57	46579.34
VPA:Contr	0.072976	0.043192	down	45373.46	40394.09	40292.11	42789.71	40673.04
VPA:Contr	0.035157	9.38E-13	up	90473.4	90473.4	90473.4	90473.4	90473.4
VPA:Contr	0.158836	0.154823	down	25660.12	26931.74	26669.93	25846.21	16141.89
VPA:Contr	0.149841	0.14521	up	1112.754	1044.428	1232.147	859.6859	1356.775
VPA:Contr	0.097752	0.091574	down	13679.55	14255.72	11381.57	14496.81	13724.34
VPA:Contr	0.142807	0.134342	down	9868.437	7199.502	10434.5	10237.21	8501.76
VPA:Contr	0.079657	0.047745	down	17408.49	16349.41	16520.87	15198.39	17559.25
VPA:Contr	0.161144	0.157516	down	7780.537	10417.35	7400.141	9586.454	6616.158
VPA:Contr	0.258587	0.237514	up	8366.856	6367.934	8182.768	6831.849	12367.94
VPA:Contr	0.244995	0.173484	up	2807.351	2606.261	4067.925	2948.319	2484.622
VPA:Contr	0.039775	0.007973	down	54456.9	53512.18	54887.51	53934.05	53988.68
VPA:Contr	0.239632	3.11E-12	up	9036.252	9036.252	9036.252	9036.252	9036.252
VPA:Contr	0.085258	0.0459	down	16499.83	15909.58	16913.82	17828.54	15421.05
VPA:Contr	0.079834	0.07518	down	11909.43	10545.61	12200.41	11553.46	12963.56
VPA:Contr	0.096993	0.081269	up	7719.318	7303.55	7466.21	6065.96	7913.193
VPA:Contr	0.247446	0.160118	up	3252.593	3690.668	2548.652	2406.227	3573.57
VPA:Contr	0.074123	0.061114	up	28624.2	32211.86	32798.91	27898.47	31988.48
VPA:Contr	0.120984	0.116224	up	14151.68	10987.36	12167.69	11551.99	15082.61
VPA:Contr	0.165696	0.122174	up	2914.406	2953.457	2374.907	3138.567	2210.224
VPA:Contr	0.044395	1.12E-12	down	74326.06	74326.06	74326.06	74326.06	74326.06
VPA:Contr	0.03984	0.039665	up	1213738	1276598	1176852	1322160	1249173
VPA:Contr	0.185035	6.14E-10	up	2158.918	2158.918	2158.918	2158.918	2158.918
VPA:Contr	0.099584	0.079872	up	8728.017	9721.135	9898.411	11220.17	9262.604

VPA:Contr	0.06162	0.059447	down	47351.66	45449.89	41984.18	45183.43	41766.52
VPA:Contr	0.114068	0.100784	up	53128.99	49753.39	43882.18	60885.21	51857.05
VPA:Contr	0.080899	0.050158	up	4125.733	4277.006	3867.746	3867.207	3728.587
VPA:Contr	0.143851	0.135529	up	6035.412	5875.968	5092.726	5245.19	7395.154
VPA:Contr	0.259789	0.074278	down	2249.832	2476.798	1947.3	2423.733	2275.207
VPA:Contr	0.095995	1.08E-10	up	10162.27	10162.27	10162.27	10162.27	10162.27
VPA:Contr	0.238944	7.19E-12	up	1480.305	1480.305	1480.305	1480.305	1480.305
VPA:Contr	0.229205	0.214408	up	4591.456	5398.53	3489.413	6734.078	4143.366
VPA:Contr	0.238661	0.196795	up	7545.903	7071.361	10830.62	5917.067	7106.67
VPA:Contr	0.201742	0.181682	down	5036.69	6657.437	5506.135	6940.11	8330.126
VPA:Contr	0.28528	0.266144	down	14732.12	8552.893	17769.81	10632.4	9110.016
VPA:Contr	0.319185	0.10542	up	1623.295	1892.965	1389.835	1526.392	1847.309
VPA:Contr	0.132716	0.129155	up	9231.602	8926.841	9574.145	6635.791	7454.973
VPA:Contr	0.271241	0.242029	up	1561.392	2171.713	1188.909	1231.225	1927.526
VPA:Contr	0.397341	1.78E-12	up	4394.273	4394.273	4394.273	4394.273	4394.273
VPA:Contr	0.154755	0.141482	up	2483.788	3000.422	2621.542	2443.612	3291.999
VPA:Contr	0.2289	0.214694	up	3605.566	1805.378	3076.516	3775.042	3038.98
VPA:Contr	0.148444	0.102669	up	4885.011	5526.505	3950.016	5028.514	5388.415
VPA:Contr	0.261138	0.082487	up	1537.1	1545.495	1271.755	1689.562	1475.026
VPA:Contr	0.173337	0.136354	up	6288.023	6253.529	5389.228	7809.439	5343.343
VPA:Contr	0.201146	0.197923	up	2124.193	1372.096	1703.432	1123.364	1485.287
VPA:Contr	0.075903	0.049326	up	4522.954	5200.481	4999.794	4615.557	5049.949
VPA:Contr	0.239576	7.47E-12	down	1149.083	1149.083	1149.083	1149.083	1149.083
VPA:Contr	0.254439	0.028866	down	1737.245	1851.135	1681.337	1781.127	1776.083
VPA:Contr	0.184737	0.170209	down	5572.286	3602.648	4249.259	4340.26	4763.315
VPA:Contr	0.176201	0.169833	down	3813.735	2255.489	2570.947	3288.895	2828.357
VPA:Contr	0.198865	0.192757	up	791.0406	961.2011	773.4612	1287.095	964.3233
VPA:Contr	0.110368	4.92E-12	down	7121.777	7121.777	7121.777	7121.777	7121.777
VPA:Contr	0.085257	9.32E-12	down	2808.738	2808.738	2808.738	2808.738	2808.738
VPA:Contr	0.234055	0.181816	up	5038.28	8320.166	5913.017	5480.149	5771.45
VPA:Contr	0.071627	0.066925	up	3758.557	4201.458	4027.541	4136.659	4461.215
VPA:Contr	0.072138	0.0642	down	8958.93	9494.784	9972.877	9987.357	8455.289
VPA:Contr	0.159079	2.47E-12	down	2360.827	2360.827	2360.827	2360.827	2360.827
VPA:Contr	0.174366	1.11E-12	down	3180.203	3180.203	3180.203	3180.203	3180.203
VPA:Contr	0.198529	0.050638	down	3528.633	3108.518	3659.396	3475.03	3317.626
VPA:Contr	0.18269	0.181322	down	2946.582	1688.582	2117.273	2059.8	2305.512

QC_12	QC_16	Control_9	Control_10	Control_11	no_13	no_14	VPA_6	VPA_7
3821.764	3764.151	16.6765	9.943367	695.355	759.4344	10.73343	15691.46	16665.24
2433.185	2510.562	2971.464	2303.091	2524.783	4526.137	1724.658	1323.278	1233.798
1876.435	1912.324	1720.627	2191.961	1865.946	2017.097	14.66741	3612.12	2778.342
1189.248	1194.48	6473.009	7187.893	6518.489	2073.734	3109.602	4138.803	2872.781
3446.332	3446.332	6108.987	7176.287	7684.812	5572.399	5799.87	4194.171	3920.937
2140.549	1760.276	822.549	744.0922	1178.014	860.5965	934.4078	7027.797	6330.3
1941.707	1621.024	436.1757	315.7821	760.987	420.4039	11.98486	7158.15	5750.946
1878.168	1878.168	2906.046	1697.373	2582.788	3031.023	2730.861	346.8282	428.0561
1177.292	1308.749	5245.409	2997.608	3934.446	1960.671	2010.79	364.3031	727.6952
935.9381	1193.17	106.4855	174.4175	98.36347	15.34947	113.8449	4829.593	4918.65
2181.174	1749.909	2356.986	3447.766	2458.015	3011.624	1981.124	1867.397	1417.159
2255.736	2239.156	1272.948	1371.112	1547.674	2449.96	1580.597	2145.377	3095.636
1723.678	2202.893	1169.045	1208.719	1358.49	2098.183	1926.789	2816.701	2454.67
7678.6	7665.028	4099.521	5367.18	5587.539	6960.725	6589.188	8143.461	8166.72
1349.411	1478.218	293.2915	312.8258	538.3518	790.9703	1069.586	2605.712	6474.261
696.0772	696.1485	810.1794	1065.621	406.7695	861.71	368.8064	174.0788	155.5495
2943.672	2962.076	4885.315	7202.9	5554.535	4359.502	2633.095	2648.671	1620.057
2477.076	2477.076	4772.474	4840.641	5730.979	5386.15	3609.079	2628.148	2279.084
1952.974	1924.554	2353.054	2586.965	3312.865	2390.566	1937.832	1806.676	1818.786
2453.364	2453.364	1943.692	2254.444	957.3075	2086.738	1231.171	232.5599	462.8833
673.4187	1359.552	3392.039	1436.553	1187.133	909.1964	1265.744	253.4283	515.7073
10129.77	10129.77	4688.598	3509.962	5699.089	8292.421	13012.72	10537.86	11122.97
2955.282	3914.162	3812.8	3529.045	3168.97	5809.944	1012.785	1581.812	1466.788
626.0394	969.44	2036.08	867.1997	1151.03	1494.685	1100.483	102.1482	186.5268
51335.27	53855.44	95565.04	57774.21	68134.4	70190.1	48912.42	40727.71	22419.81
50145.17	48073.69	89378.6	56633.21	68719.04	61658.34	41974.8	33854.22	23206.38
33869.17	34140.47	46480.06	33838.25	40165.54	47318.75	36976.11	24555.61	21999.01
9934.948	10343.66	8538.935	10703.71	10754.39	10446.11	7080.95	5862.136	6923.093
6240.803	6662.292	6319.535	4752.244	16432.64	3472.595	17787.85	780.5563	2628.148
5637.766	4273.727	7019.611	5428.125	4666.44	5217.777	3380.609	2931.66	3608.562
6453.846	5605.181	7239.278	9430.91	7571.376	5708.602	5183.991	3700.075	4378.143
9685.238	8167.411	16773.64	15068.93	14114.71	10880.71	9987.499	10436.46	7830.739
22223.39	22058.63	42878.55	20059.87	39736.94	31234.01	25593.98	10290.26	6428.444
21051.77	20698.82	39659.29	17692.5	35456.18	26870.88	26622.01	10950.25	7382.694
15278.89	15280.6	23325.38	11798.06	25509.68	17862.54	19638	5973.873	6229.427
26323.73	28411.38	44724.6	28832.92	50166.09	31770	36173.1	17903.02	19645.35
8034.014	6107.811	14881.76	10553.84	8620.676	10608.5	7636.09	6692.231	4534.098
4607.544	4410.164	5934.929	5836.854	7402.292	6970.609	4970.063	2551.898	3270.344
1866.596	1866.596	4784.882	2737.578	6240.91	2573.256	3570.916	1107.815	611.5849
2323.779	2158.216	4395.046	2744.687	2985.787	1703.981	1727.11	2152.29	2084.178
7124.84	7124.84	1710.237	2949.581	2706.924	4197.772	2698.089	4578.311	5665.23
16754.4	17871.83	40307.2	60254.03	36208.17	37019.66	31266.7	16233.56	23269.12
12523.23	14103.43	56582.99	60146.36	34967.13	27801.88	24118.02	17490.25	19812.31
1051.435	1119.708	609.2127	721.6719	644.583	1571.036	1232.361	1271.982	848.8902
9006.807	9566.658	13756.47	14977.36	18994.34	8126.075	23394.63	10463.51	7655.651
5476.946	5349.39	8637.528	13854.56	15177.25	7020.375	6105.578	6100.13	6032.519
74639.74	74639.74	59758.23	59860.1	110058.3	46840.46	50446.06	147067.4	144519.2
1241.912	1223.822	1431.8	1398.499	1257.114	614.1637	710.6469	1940.133	2463.687
8083.471	8139.813	9695.05	12609.81	7790.274	10803.5	7955.418	4867.465	6425.141
3863.394	3963.331	5578.85	5011.292	4029.98	4960.771	2479.11	2685.614	3125.349
1910.078	1910.078	653.5676	769.8813	413.2454	1685.791	1721.345	1491.622	3285.145
6290.094	5778.385	8876.153	7633.17	8325.417	6989.753	6746.278	2932.133	3244.85
13611.7	13611.7	2260.131	1264.27	2647.158	4905.257	7649.051	6227.901	9627.612
3716.689	3716.689	3948.18	4435.124	3772.329	7165.558	5195.718	2648.98	2510.496
803.8273	1513.629	2068.527	2332.604	1697.452	1502.429	1355.557	850.1476	1134.908
1497.355	1497.355	509.1271	423.8136	458.8288	1047.081	1202.336	1700.273	1094.807
729.9208	719.2284	454.7655	692.5167	441.7284	567.5647	517.9711	1210.599	902.8331

235082.5	265570.4	1289420	1038017	613716.2	243853.5	277051	118817	183536.7
81900.3	81900.3	310090.5	253174	158284.2	73168.96	85757.52	35784.6	56889.29
3610.71	3616.843	5449.135	5234.094	7238.04	4610.489	4503.911	2989.989	1903.483
1306.497	1306.497	1739.7	2036.32	1640.239	2055.406	2003.928	1033.968	1068.252
3143.438	3385.636	3520.642	2823.518	3459.292	3465.284	3624.988	2095.812	2089.291
3937.119	5153.556	2082.317	3470.287	3064.216	2985.49	4468.023	4965.473	4505.513
1387.789	1395.024	3539.837	1830.392	2535.701	1450.48	3015.845	951.8946	1175.505
1467.076	1467.076	442.4527	472.7245	180.7612	1907.892	892.8635	2886.588	2186.032
3170.358	2831.983	2889.065	3058.46	2471.195	3319.16	2176.124	1536.779	1646.603
1425.647	1775.611	3447.843	3782.993	2771.165	1908.684	588.4692	1799.1	1804.462
9402.662	9670.682	5465.545	4864.215	4416.551	9668.74	11605.57	7530.327	12247.32
4930.238	4929.963	2524.076	2158.067	2984.282	6536.536	5365.325	3672.598	5152.521
2286.667	2138.233	114.4471	56.52258	11.5351	7.962969	12.43496	389.9706	2554.734
18650.56	17159.41	33872.45	22911.86	20915.02	17049.54	19363.94	14393.45	12187.24
7499.618	5549.482	10229.38	8508.47	8355.83	7011.807	7260.15	6017.602	3887.131
1605.708	1623.319	1625.559	932.8755	2119.129	1324.845	3452.707	505.7509	543.7575
2809.487	2809.487	1151.884	641.3744	1900.802	1395.5	2705.959	3753.705	7798.854
6556.295	8177.854	9103.903	8522.485	10224.51	9436.134	9034.064	6781.439	5863.752
1653.653	1222.184	521.6826	657.778	769.9766	921.478	17.62631	3468.701	4150.497
2952.555	2952.555	6204.226	5361.697	4641.236	4454.179	4302.078	3464.579	3684.955
1385.259	1423.831	1184.122	1078.666	869.3545	1097.466	1509.924	1819.562	1658.738
3672.675	3672.675	479.8009	782.6565	2150.522	1014.029	649.7579	12018.7	11352.91
3244.186	3242.628	2136.677	3006.037	2083.734	1876.008	1985.529	8834.042	4804.092
1826.406	1982.962	3324.76	2939.509	2280.309	1975.119	2618.88	1871.884	1667.793
1379.456	1404.118	3875.29	4649.976	1793.59	2980.346	3354.28	1135.375	580.5434
2211.429	2286.179	1176.231	1615.695	1705.48	886.6218	1386.119	4157.364	3588.926
15276.35	15634.64	28272.01	29058.74	34071.28	16943.42	16262.09	18557.41	16025.51
7898.995	7898.995	4234.38	4037.923	3829.2	8313.737	7961.531	7265.33	6545.395
1024.466	934.8587	1244.336	1486.734	1356.057	1621.086	1048.708	944.9652	838.5688
15272.95	11034.76	21216.17	15047.45	22842.67	28250.68	18357.33	6834.741	11698.28
1726.254	1719.878	779.7416	427.3212	817.385	1018.243	2233.494	1908.863	1547.227
30132.83	29064.83	60731.01	43874.49	34232.35	34370.38	31219.18	9952.05	11585.52
9121.26	9510.939	16958.24	12445.74	10769.01	11482.79	9642.733	3239.417	4692.941
2506.522	2720.481	888.5142	1237.915	1656.351	1684.158	1845.793	9893.987	7832.888
1315.625	1311.626	2513.8	2739.3	1483.083	1711.715	1729.04	637.8256	639.6873
5437.793	5071.134	3518.361	3601.134	3811.14	3446.254	4167.993	7815.72	7552.232
20133.65	20080.12	2172.526	2307.179	13277.13	19045.16	9079.818	19844.74	72642.3
8074.145	8074.145	11321.06	12428.01	7854.926	10851.26	8386.839	7805.877	5315.906
2507.665	2533.484	3653.968	3501.818	2564.143	2485.789	2474.633	1955.1	2144.145
16452.84	17148.97	24368.7	16798.78	15668.55	22059.51	14733.76	9559.466	11068.73
30912.92	31324.66	63526.53	40282.47	40130.34	38789.68	31727.55	11122.23	14429.82
12112.15	10856.15	19424.03	12282.68	13244.51	13308.44	9115.34	4095.287	5101.048
1913.575	1243.452	1678.109	1848.962	1273.038	1890.51	911.6205	1121.342	903.6434
23024.07	23024.07	6021.847	8195.442	14745.38	10869.57	11927.83	17683.4	40997.99
4232.681	3198.258	4253.153	2861.274	5242.71	2935.299	2173.246	1001.64	1533.095
1371.9	1371.9	3760.412	5806.285	3319.729	5172.628	3599.288	851.193	1326.654
1249725	1333895	2570958	1607711	1738402	1688761	1331863	364369.2	587033.2
341692.7	341830.7	592983.6	388572	431728.7	446039.9	332744.1	97107.09	146785.1
12707.82	12707.82	8921.821	7776.853	7630.844	13300.72	12155.96	11829.59	14038.76
26270.03	26270.03	870.0033	1309.059	6607.984	9445.991	8801.935	8566.412	51094.82
70952.63	70952.63	141371.8	87532.61	95454.22	89122.37	63891.64	27484.38	41701.25
8761.966	7801.873	8163.57	7719.809	8541.77	8530.831	6158.395	5487.6	4283.06
11676.41	11195.17	16709.97	12054.66	15179.29	11991.71	18565.48	6054.366	3002.207
6043.406	5876.526	10458.31	6712.466	9494.313	6203.517	7958.237	3395.662	5717.526
6339.245	6313.772	7310.353	5523.665	5917.25	6109.525	4834.603	2387.76	3181.174
2695.252	2655.994	881.886	1262.362	2378.494	1242.396	1555.865	2694.296	4852.321
1443.547	1397.117	3052.618	1732.128	2485.143	1204.262	1551.417	1406.235	915.7353
4084.724	4084.724	1487.803	1490.23	1975.838	2156.253	2390.712	4394.048	3465.678

1408.634	1408.634	5091.16	3606.417	3854.609	2533.436	3616.489	1666.399	2677.412
1534.018	1539.497	4131.75	2553.143	2768.053	2431.63	2425.395	1401.803	900.243
1682.992	1682.992	1892.82	2425.288	2085.175	2107.374	2526.122	980.7853	586.8346
883.2689	883.2689	326.0452	430.5075	509.2175	695.6499	828.8439	1323.411	798.5887
2343.112	2343.112	1294.872	1850.373	1725.925	2250.112	1768.814	697.3527	586.7923
4996.521	4996.521	2646.878	2946.515	3348.935	3673.595	3501.284	4752.638	6074.958
868.2093	832.4304	271.8873	312.1181	509.4138	812.2062	951.4483	568.532	850.0132
2122.99	2261.696	2189.134	2274.928	1810.219	1879.842	2595.48	3682.544	3749.821
145844.9	147145.2	285168	221248.9	192546.4	182775.5	134018.3	45393.49	62426.98
45599.49	44187.21	80130.39	60631.1	60586.5	53516.07	41152.29	14622.57	19267.03
203916.9	204455	391777.4	235578.4	355497.7	232815.7	328555.4	132350.3	155477.6
60909.84	62769.7	110639	73927.24	108818	71564.19	91450	44443.48	47065.24
1347.09	1375.863	1627.967	1921.882	2618.127	1733.365	1575.743	979.5934	850.6678
11746.32	11746.32	5345.375	7659.377	12082.38	8401.204	14017.52	14566.38	21841.39
1146.46	1194.32	1773.355	1640.88	2173.819	1186.8	1816.312	786.2368	667.8165
2135.228	2135.228	1013.354	1008.579	1214.703	874.3302	1950.898	2108.68	1852.416
4347.037	4927.596	6903.66	5363.608	5939.503	6562.597	2928.755	3661.675	2293.194
5872.944	5047.878	7269.779	6679.205	6996.548	6339.275	4702.049	3278.175	2998.37
2246.925	2318.485	3579.955	4096.568	3505.031	2777.41	2688.669	2506.023	1990.803
22241.8	22241.8	23467.02	47796.57	26020.94	31034.89	28948.88	15843.21	6037.219
1752.492	1752.944	3145.693	3962.256	3245.684	3086.176	3745.541	1253.955	770.7689
1774.685	2472.961	956.6474	1483.639	1914.191	2831.473	2983.067	2845.363	3784.897
1283.071	1270.248	794.2572	849.8635	1117.848	1364.643	1368.946	1804.082	1630.911
32552.3	32387.12	5746.931	11724.01	25143.16	22344.8	17645.91	30543.71	133730.7
11889.5	11516.2	19514.85	20227	13997.18	13855.75	11853.52	9566.901	9539.476
24844.53	24844.53	49863.25	36455.91	41812.4	26791.41	35924.17	15698.85	16142.3
9527.001	7916.615	15962.37	12337.37	14966.29	9338.606	11291.31	5259.14	5156.942
4561.675	3169.283	5044.787	2850.998	5958.333	4742.102	5580.298	2315.8	1079.722
40343.27	40343.27	71707.64	41197.27	69340.28	40950.26	56578.32	28976.8	9229.348
13942.16	13713.23	23158.89	15542.89	25648.72	14456.12	18427.69	9655.888	4613.482
4020.748	5390.077	3011.122	3311.171	3010.973	5214.051	5741.23	5860.135	5509.53
19839.85	19723.6	33835.05	25467.9	29533.92	21948.54	31305.99	13384.74	13817.44
7374.153	7278.105	11811.41	9108.981	13221.63	7984.785	8808.095	6047.688	6971.068
27788.68	27788.68	21252.69	16860.77	30071.52	23291.08	12360.06	31990.37	56945.15
4484.785	4484.785	3112.778	3272.39	2865.223	3178.064	3427.705	5100.633	6786.819
2414.929	2414.929	1767.981	1251.518	1973.78	2882.234	3297.189	4029.691	2413.189
9557.264	7817.529	16501.21	17936.43	17363.63	11530.81	13060.42	11774.3	10123.36
3146.126	2952.926	4828.641	4247.048	5359.005	4463.592	3835.612	2761.202	1913.074
2283.678	3049.683	2171.955	2651.687	2227.201	2500.056	2960.412	3403.76	4008.112
3279.907	3381.137	6098.065	5318.3	5126.62	3846.839	5194.157	2013.408	2241.197
3482.039	3002.974	3156.249	2645.448	2342.859	3197.733	3192.468	4731.308	5058.811
10192.06	10192.06	42543.86	53625.81	94527.26	57395.66	37979.89	12242.41	19375.15
1784.671	1941.919	3343.021	2446.793	2750.592	2615.806	2202.837	1474.296	1914.12
54464.81	52119.92	38999.86	40541.05	64188.93	33906.1	37326.48	90730.34	73531.6
23865.58	23865.58	20883.76	21697.15	21989.84	28047.96	18881.88	27693.56	36472.53
946.0188	920.5849	3180.609	2417.808	1429.627	701.91	1161.001	583.962	334.102
4649.331	4480.271	1465.544	1314.493	1872.033	5165.886	2618.294	4792.896	4390.168
6076.996	6076.996	7338.31	5393.633	6326.271	6526.395	6222.025	9022.264	10734.39
6430.177	6431.766	11259.45	10509.9	11940.65	11758.05	6946.583	5244.734	4859.594
4092.355	4163.512	7360.012	4795.659	6307.353	5241.129	2980.693	917.2572	1236.274
1104.88	1573.049	2044.809	2401.289	1574.705	1504.322	1132.754	1328.285	1078.096
8517.242	8471.363	7459.171	8903.344	9764.845	13491.91	8461.953	3725.353	3434.657
13938.74	11629.6	14089.75	17015.95	18044.7	18959.26	11943.47	9768.838	6915.026
1572.975	1571.472	3157.78	2926.908	2794.169	1644.861	2132.578	1701.629	966.0397
40364.04	41090.95	22252.35	42735.83	56287.66	15071.78	36256.94	88307.31	195663.8
1568.37	1123.117	446.3162	640.4008	893.8891	781.2978	588.3922	2847.102	2043.034
17242.27	18235.61	20784.11	13670.93	25281.89	25022.52	19874.52	10977.77	5748.906
1868.968	1868.968	1958.338	2507.014	2747.788	1066.894	2312.807	1306.143	1740.42

1984.72	1895.394	3716.078	2858.795	2249.218	1938.847	2058.036	1843.866	1484.993
4349.416	4132.97	2726.112	2722.414	3633.589	4155.008	3927.845	4333.713	4414.258
749.0695	1181.381	468.4645	369.6961	466.6123	559.9188	627.1082	1224.83	1298.352
18136.02	18136.02	10373.02	10281.58	14299.61	18025.18	17987.13	21537.45	17919.48
13426.42	14028.78	10356.6	8911.37	12146.58	8742.182	15014.2	15871.49	25928.03
13491.35	9890.592	5703.214	6118.463	8071.92	9729.651	8984.108	13877.92	11436.95
1040.298	844.8289	712.6334	464.8715	568.0183	1193.078	891.1166	1075.777	1075.64
9282.341	9298.733	4506.224	6689.433	12019.1	4043.134	5731.338	21686.38	31583.84
2808.942	2607.347	2649.997	2870.878	4294.375	4842.834	2891.627	1456.915	1172.735
1038.682	1038.682	705.9086	512.9627	653.4171	1561.028	564.796	909.7875	1091.117
14386.65	14386.65	8504.143	7839.647	6820.863	8147.342	10579.58	30163.96	19805.32
6309.476	5217.2	6910.353	6066.378	9246.012	5791.878	4968.401	4829.735	2783.9
1312.944	1307.602	588.5456	862.1866	826.1205	1098.292	836.9623	1111.499	1918.988
1397.417	1592.673	2015.977	2608.259	2114.248	1308.175	1680.264	1178.895	1310.901
12236.49	11042.09	17943.68	18520.95	11673.02	13186.06	9841.526	7726.212	9828.44
761.0991	730.292	1635.588	1731.428	1231.797	872.5202	982.207	545.3028	678.1856
2741.819	2753.636	1264.918	1419.167	2806.259	2972.6	3723.113	3505.377	4500.933
12719.58	10566.38	10344.18	16586.09	11865.32	15894.46	8127.376	5228.172	7518.621
16237.83	16358.19	11013.01	11896.63	11996.26	22394.94	14970.35	18058.52	24356.6
8820.422	8857.26	4238.545	3559.124	5749.312	5450.767	9804.739	8913.757	11288.32
11493.02	11278.95	9408.093	10665.04	9052.149	14078.7	9351.511	5432.797	6026.855
6805.054	4917.604	6190.907	5764.208	5102.221	6419.477	4354.768	3020.021	2277.588
2707.335	2707.335	2745.831	2809.186	1991.013	2860.484	4344.078	1177.583	1847.774
2609.022	3303.415	1784.862	198.4572	220.0062	63293.24	230.5455	4105.245	20359.96
2160.84	2160.84	2851.298	2149.052	2445.409	2642.12	3448.743	1413.35	749.059
4755.314	4774.423	6927.777	6866.195	6902.431	7804.087	3402.466	4347.472	3886.585
8361.754	8569.644	11506.69	16550.72	10642.38	16653.68	7537.594	8178.948	6951.308
18727.26	18800.36	21199.78	24723.6	22701.72	28948.73	23078.29	13680.26	16195.17
2879.235	3738.819	1167.695	719.187	1682.631	1119.919	2244.562	7754.764	7362.432
9092.276	10648.22	11142.05	22144.47	11636.9	12631.21	9769.431	5580.832	7628.881
14566.89	16491.8	21957.44	19823.2	18435.66	24242.45	26429.85	11991.84	9006.872
51829.42	51830.05	56954.46	70125.53	56176.45	72387.16	42162.57	23955.84	35277.85
13359.89	12730.66	8105.674	6304.95	10943.16	11876.11	8427.883	14536.69	26466.94
148027.8	158360.5	204010.8	221852.9	164877.5	195076	147260	81395.33	60720.41
3128.1	2989.85	1541.612	175.601	200.6729	62375.09	237.7036	4650.59	16105.62
25712.32	25712.32	30998.99	35658.56	38787.05	39671.6	23655.08	15458.2	18470.64
7202.28	6761.897	4467.775	3207.452	5331.825	4345.678	3758.255	9099.059	10857.55
67952.62	77385.46	90850.2	99675.56	85747.21	87573.3	72000.77	42213.12	30500.81
2252.639	2250.04	2378.727	2393.462	1522.235	2216.494	1846.747	1043.05	1341.117
1785.443	1803.509	3415.129	3295.408	1810.954	1897.207	936.9046	916.3493	1308.607
167451.8	167451.8	211133	242895.8	186024.1	240619.6	135189.3	80179.9	103769.5
73258.05	71818.69	91548.8	109382.4	86819.21	116846.4	61438.91	39005.4	50425.31
20431.35	20470.36	25834.51	23270.66	22276.46	28686.69	15352.48	8707.986	11853.53
7413.404	8934.772	4035.169	5837.688	6202.423	316.6401	182.0545	19155.64	8961.729
27053.76	19475.2	38838	38960.44	24863.48	34679.36	21280.24	14712.31	9735.586
11463.14	8886.493	18308.06	15404.63	9610.268	15545.09	11756.25	5674.303	5889.018
3700.568	2244.631	3711.178	3533.718	3383.132	3170.414	2713.257	2326.261	1350.941
350076.7	350737.1	505889.6	648331.6	346905.1	511597.4	362729.6	247581.4	197535.9
6920.734	6920.734	6264.993	5608.895	7675.696	9012.234	9513.604	3522.878	3769.872
165768.7	156164.5	239707.5	284425.8	159798.8	225502.4	164006.1	123981.9	88204.21
44889.11	42699.41	62472.06	69835.92	40590.81	61043.16	42497.58	32244.97	23634.56
276898.1	277802.5	368819.5	451527.2	286336	383492.6	280054.9	267012.5	155046.4
1302.811	1302.811	781.4365	427.6591	515.8483	561.343	514.7296	4364.955	4903.861
2473.735	2361.28	941.5701	864.9429	1565.12	936.6156	1488.383	7966.242	7066.945
3082.531	3082.531	1418.221	1644.897	2872.122	4495.595	3557.456	5400.921	3992.915
840.9123	842.5293	176.2509	208.0758	298.5828	264.1455	393.6321	2035.142	1849.691
2702.238	2702.238	2089.859	1804.924	1485.566	1712.668	1255.794	2986.27	3811.974
16504.13	18409.05	14404.4	9598.475	13842.76	23993.21	9787.483	18401.62	23103.9

919050.3	917775.3	1243208	1100068	775718.9	1343270	688483.3	376027.2	438791.2
51590.12	38047.43	54530.98	59576.94	46514.02	69850.58	42826.38	24816.37	31388.02
392194.8	392194.8	490635.3	417504.8	311816.8	540855.9	291921.5	160136.4	184512.9
4200.501	4220.85	418.6197	986.2347	2307.683	1759.329	799.2731	12560.3	10677.66
947.4817	1243.797	567.8887	477.4868	911.4289	844.7324	1143.608	1450.209	934.3485
206292.7	197978.8	335656.9	239859.4	196107.6	321784.9	174421.6	112421	112323.3
95407.67	95547.07	144105.4	106787.3	102300.4	142468.9	69916.24	52520.98	66353.14
326755.2	329298.7	520334.7	413042.9	373838.5	512651.8	322153.2	193481.5	256645.5
147340.6	144867.2	231900.8	168152.9	174369.6	223284.6	117173.9	95145.41	106520.8
37155.42	44862.32	52861.5	43675.93	41465.02	54185.64	26050.48	22185.1	27923.62
8906.985	10312.78	12844.77	18948.38	10380.34	10394.12	7847.253	8735.723	5998.607
2844.224	3931.037	462.565	155.1525	1191.712	1680.766	2053.865	4818.871	18032.38
12653.62	10444.17	12413.08	12209.6	12884.03	16865.62	11950.02	8168.671	7974.09
9211.016	9209.581	15691.41	15605.26	22220.33	14004.22	6943.107	10573.99	6860.095
6575.829	5771.714	5035.85	3856.45	5134.162	1151.663	606.5765	11197.32	8718.584
25439.05	25452.34	61604.97	54680.97	32214.25	26803.58	34951.89	28534.22	19077.53
3274.803	2516.168	1803.929	2088.557	1639.472	3351.545	3526.013	3978.537	3555.501
13573.97	13580.03	12912.81	14843.35	11803.09	16650.25	9598.554	9793.776	8955.315
44394.3	40098.88	31029.28	45749.79	33606.68	50499.5	31934.63	21055.13	21833.41
2138.067	2095.957	754.1241	652.7208	1572.946	2199.389	2887.317	2691.797	3148.415
5243.836	4642.235	4898.217	6297.604	6506.545	7062.159	7847.402	9821.559	9039.292
962402.1	917622.6	1424279	969298	1102428	1186863	947180.8	558980.2	689132.3
421172.5	438913.4	602458.8	448448.6	495286	551871.5	432005.2	259934.3	315149.9
5881.223	7470.923	4013.071	5450.082	2083.359	60.539	98.63368	18001.1	8764.097
31833.43	33011.32	32992.49	27301.34	41417.64	34059.3	51358.77	19935.77	17543.3
28262.58	16119.45	29986.53	25895.18	29444.71	19438.95	19108.58	10507.28	8152.966
74160.17	74160.17	133576.1	79021.43	77725.29	139585.8	58709.96	37497.29	46588.42
667365	648448.9	691276.9	686737.7	943555.6	818347.8	493220.8	428837	554965.9
198332.4	197505.2	235733.4	172914.4	184181	261146.5	139960.5	88749.84	111515.8
312197.2	305864.7	311553.7	319388.9	448901.1	383747.6	236292.9	202690.8	250488.7
88074.49	88450.69	122403.9	99004.07	107026.7	144741.6	73191.71	49139.34	60729.99
36037.36	24393.19	33795.55	42232.14	44265.75	36303.96	19316.96	16107.73	17499.52
23531.46	27923.82	28233.74	32455.78	31980.79	36746.77	22497.16	23040.71	18733.4
13489.35	13409.95	19929.73	17531.27	17934.2	39986.54	16019.76	11414.53	8476.417
9800.028	9915.199	16905.33	11422.23	12105.32	13339.92	20008.29	5083.91	7121.115
4291.899	4421.025	5812.72	5691.743	6998.259	8666.008	4143.479	1760.802	2333.968
1696.417	1367.989	186.472	382.0593	1150.38	713.8953	382.6806	4716.219	3837.529
2553.165	2863.045	4652.488	3458.498	3575.165	5381.963	2541.286	1264.341	1048.49
4209.082	4894.707	7707.87	7015.225	6206.509	5637.646	4199.549	5213.054	3664.898
12685.43	8944.621	26891.2	23513.35	12379.26	339.1695	441.2028	11893.28	4372.925
10334.65	9678.35	12857.73	14624.62	17112.2	15051.53	10448.85	9901.904	7410.831
4204.028	4730.409	2237.766	2232.787	3907.287	1706.082	59.869	9730.068	7505.997
4408.577	4408.577	7539.134	6421.269	6131.553	5852.54	9995.616	1929.875	3532.502
19641	20277.43	29481.54	15882.95	34695.61	32382.79	24391.1	8969.095	12670.9
371127.6	371113.1	489915.3	296075.8	445700.3	456968.2	365894.5	201626	278460.4
17352.67	14572.78	18801.55	17790.76	25286.98	25233.01	11900.55	13608.67	12236.08
1116.358	1600.607	667.344	720.8079	1159.874	2099.04	1365.292	1873.575	1538.081
184070.9	184070.9	244453.6	146367.3	204841.9	217713.3	174221.5	97898.45	131125.7
9232.82	10497.75	12037.28	12372.05	15268.41	16751.97	8167.582	6785.382	5769.818
22231.2	22231.2	30851.9	30312.77	28620.8	25269.71	22495.56	22846.29	18590.15
10632.4	8891.349	12776.62	13820.73	16324.63	14165.72	7093.338	5934.245	6918.535
10317.22	10317.22	4024.586	4800.121	4093.449	5960.133	7913.206	6951.272	6854.248
69347.74	71249.41	102504.8	78859.18	104783.9	101245.1	67100.68	48427.02	48217.57
207817.9	203014.6	368407.8	347349.9	271914	315167.8	200916.7	165450	184370.5
7260.96	8178.648	761.0563	969.9512	3858.152	4706.002	3828.374	6254.128	18582.94
106725.9	106591	205333.8	174268.5	141757.5	179127.3	95633.28	85499.51	98607.43
10007.45	10247.69	27610.1	45722.67	56819.32	16615.71	17855.09	20355.75	13248.54
43473.98	40509.29	73944.23	48763.68	56341.63	43698.08	39177.93	39129.53	29143.59

9160.207	9207.906	20275.56	18088.4	12666.11	12336.07	6220.821	8782.049	10192.07
12702.26	13273.69	20410.57	14803.95	10123.04	7515.451	22200.3	3109.053	6647.752
10468.34	10468.34	13097.14	16300.69	20626.23	12583.4	13305.16	4803.65	6942.633
78684.78	69121.56	112913.1	93640.66	69794.24	75450.35	158704	27479.28	42365.76
4417214	4417214	6601394	3523584	5488792	5110414	4932970	2267909	1424631
1932296	1913086	3249186	1690273	2704061	2498075	2291474	982365.6	759928.3
5729407	5930357	7001112	5962726	8804345	6663894	6919480	4227600	4745009
2459059	2599139	3007998	2590863	3775835	2845201	3041931	1918046	2074992
958942.3	880769	1293063	1320929	1017369	1317750	908228.6	680598.9	591282.6
56148.32	56148.32	90396.68	82862.46	72400.86	77574.42	52367.87	54117.28	49431.32
476912.7	441962	640503	623998.4	498497	639197.9	471642.3	341175.9	306644.4
26078.82	26072.2	39965.22	38886.32	30965.8	26802.38	30894.28	23910.32	18957.12
144973.3	136980.5	186350	192590.9	148259.8	189003.8	144563	109018.5	104878.8
1684768	1684768	2317679	2002034	1954778	2066250	1556604	1596005	1201064
19359.93	19349.6	28679.16	29319.21	23956.43	29617.96	22210.03	19520.08	11827.21
837612	799936.6	1163698	1015432	973439	984217.4	757481.5	800956.9	607864.4
251240.9	225359.7	333111.6	327827.4	294716.6	293767.6	204768.1	247316.1	192968.6
104874.1	97889.19	120752.4	72168.77	131446.6	142869.1	81891.36	57231.56	44514.46
26020.94	26037.44	26573.44	23628.51	26626.47	27770.22	24626.18	16879.25	17176.58
23854.2	21830.87	32941.88	19866.61	31294.7	23870.45	21917.91	13740.64	14988.73
7349.523	8079.548	16246.96	12775.86	9920.475	16759.11	7082.026	3206.198	4849.271
55798.83	54569.16	88726.54	78969.44	55376.46	77342.38	53261.09	37708.12	46659.45
34111.61	35831.08	52215.76	45157.94	35579.41	50316.77	47970.47	22662.05	26234.57
991.3103	994.1729	31.59456	34.60161	21.93471	23.47909	37.12896	3110.357	5976.283
52871.52	52955.84	98238.57	55242.55	96434.54	79549.83	59362.79	51501.15	25131.32
4617.612	4955.377	7731.257	2800.384	7145.178	5728.047	12077.71	1517.819	2245.506
103985.4	113037.2	95354.25	145320.9	133485.7	103950.6	121707.5	70367.83	50130.35
58379.55	61233.18	58256.15	86902.49	70291.17	60345.7	70677.16	39950.89	28842.63
38954.7	36721.32	62982.88	24350.37	42611.38	43477.76	79177.74	10068.23	20577.85
15110.61	15662.95	23873.59	16705.82	20125.24	19819.55	16913.06	14429.65	13414.37
14456.36	16101	28506.53	22816.06	17249.11	11783.93	23243.61	15115.04	8772.13
9087.544	8565.989	12615.67	8630.667	11186.91	8790.906	8357.862	7932.066	6338.413
18787.29	18790.54	18766.04	15815.97	18573.66	21575.84	15082.01	10159.17	11082.64
76190.75	76205.98	87827.81	98157.15	84658.52	94244.26	74799.24	63231.08	51979.83
50440.68	46466.6	64041.61	37609.79	52959.93	64470.43	44410.21	27472.92	19273.26
42729.96	42032.58	47818.14	55584.22	44095.88	56618.71	40584.46	33434.32	30134.04
90473.4	90473.4	74778.07	60908.98	73371.99	68397.88	95844.19	86812.34	131354.1
26412.12	24735.48	22220.06	32432.15	28731.15	33648.31	21421.43	17419.6	20870.25
1004.643	1150.651	44.19793	51.64239	20.56515	21.03809	21.95334	2765.087	5665.088
15035.53	12571.57	23015.26	18942.58	15552.88	20726.47	12320.37	10947.04	13842.9
10361.82	8468.033	14636.89	11637.96	11359.21	13036.55	10614.55	7460.425	7820.789
16292.24	16856.8	18050	21266.12	18550.05	27323.15	15729.05	13374.31	7625.926
7930.079	8789.617	9171.472	6909.386	10160.45	13508.58	5063.762	3847.511	3788.243
7880.036	7685.353	3367.179	2700.486	4947.592	5790.025	10050.68	7167.986	9316.744
2983.601	2943.413	874.5887	214.7382	22.27787	66.89397	499.6479	18924.77	16761.77
54192.74	54182.42	86939.03	44575.87	93430.7	106914.4	59068.26	19204.4	24345.65
9036.252	9036.252	5198.933	4158.493	5386.258	3975.153	4972.065	8556.215	12058.63
16526.11	16510.44	33212.56	17580.41	26972.52	28740.99	12821.64	8952.044	8562.322
10636.81	12234.98	18805.17	13624.77	14036.81	18547.72	10200.11	7001.133	5232.801
7417.806	7271.619	3019.566	2799.582	5925.535	7682.943	7088.886	9552.908	13491.67
3387.202	2933.194	1503.34	164.1286	178.6559	49553	177.2288	3865.23	17354.15
31162.4	30087.41	8815.441	12508.58	25387.1	23075.64	24640.32	26542.11	60898.94
13431.74	12056.32	3810.631	6809.829	7591.076	16143.56	16783.55	17526.81	19109.13
2895.759	2712.437	2113.817	914.882	1889.049	4528.588	1937.331	3674.658	5380.296
74326.06	74326.06	122604	57156.78	112220.7	153040.6	75068.35	24529.07	27544.15
1203143	1261414	651996.5	810147.3	1314187	791838.4	789566.8	1414624	2550174
2158.918	2158.918	1028.061	1106.736	1291.611	1507.271	1885.699	1891.446	1562.332
9362.941	9696.083	3620.446	4629.753	5675.357	10507.66	10269.12	10704.48	14584.99

48674.28	42987.25	73841.31	37518.2	70606.39	92786.1	40621.73	15429.92	18832.03
48575.85	52679.08	26832.17	31310.9	52227.83	30330.71	34904.04	59108.96	90132.26
4197.184	3958.182	1884.548	209.4648	231.8141	66560.78	242.3701	4649.716	18536.64
5252.462	6097.358	2077.058	2839.107	2969.473	6063.141	5117.871	4122.386	4121.71
2271.812	2263.506	3972.121	3227.52	3038.816	4297.207	2804.047	1467.838	2069.351
10162.27	10162.27	5399.672	5925.378	8083.656	9786.99	10425.27	10508.56	12004.27
1480.305	1480.305	215.0393	185.7672	295.3385	333.4443	648.2744	927.8721	5455.146
4472.004	4912.383	1577.334	1250.788	3022.374	2013.348	5049.413	4068.751	5894.042
7740.295	7644.588	4687.271	4381.681	6408.414	5623.791	6445.779	7649.972	8716.525
5302.099	6423.256	11381.35	5023.297	7235.716	10272.27	6013.357	2501.897	3336.41
11903.33	12445.17	20884	10798.29	27266.71	21153.07	10611.49	5427.541	5866.626
1638.019	1641.787	445.35	379.8969	959.5875	927.8235	772.3428	5488.761	6061.109
9362.369	8291.464	2157.033	1764.594	5415.873	6965.23	7535.696	6561.561	14069.02
2004.42	1379.486	253.6921	252.9572	576.5644	937.5572	1302.13	1803.041	7489.555
4394.273	4394.273	2377.072	2470.992	4284.616	7044.265	3737.425	7278.376	7374.523
2213.738	3015.991	1774.05	202.8813	231.3258	70400.31	263.7473	2782.257	19869.11
3254.802	2697.016	1652.942	187.5092	213.3092	65730.92	248.9814	2567.04	15689.17
4881.629	4895.751	4256.573	2900.953	5787.248	723.0423	18473.07	7459.02	9395.845
1500.362	1494.692	274.9098	293.8694	481.7815	463.9696	537.0763	4242.446	4604.283
6731.413	5899.68	2206.49	1857.365	4719.102	5027.888	5429.081	6742.87	8916.334
1710.79	1565.105	139.1422	281.8471	158.6465	330.7068	501.2948	4652.926	5544.211
4831.209	4844.273	4128.927	3323.71	4943.762	2856.992	4501.838	5983.282	8945.441
1149.083	1149.083	2458.373	2274.865	3301.283	5109.793	2139.731	404.5192	706.9678
1764.937	1763.231	1618.194	1489.415	1398.012	2815.162	1732.849	436.6595	738.3191
3548.36	5174.514	5459.513	6296.374	6571.701	5414.393	3856.261	3972.772	2435.083
3023.401	2895.827	3958.878	4390.809	3276.19	4099.193	2610.302	2214.093	2101.976
790.4464	970.0111	98.09178	203.7149	117.1185	89.89943	120.2685	4886.324	3698.692
7121.777	7121.777	9660.207	9344.963	9802.859	10554.91	8289.28	5522.757	6024.676
2808.738	2808.738	3094.569	3663.507	2284.658	3666.393	2672.233	2051.579	1504.268
5370.151	6521.181	39.18537	84.43257	10.05087	538.3629	9.895275	43280.95	28382.85
3690.79	4239.36	45.73615	95.59361	74.33027	757.8977	746.762	18575.93	15491.2
8851.712	9731.044	14570.2	10380.7	10953.18	8926.74	10087.09	7087.142	4833.264
2360.827	2360.827	2620.501	3857.236	3454.84	4409.979	2707.838	1554.476	1221.565
3180.203	3180.203	6594.042	6714.509	8131.595	5653.389	3612.935	1471.573	2773.504
3413.583	3415.945	7389.379	9482.168	5001.238	6134.527	3003.869	3968.495	3320.57
2564.375	2049.382	3751.18	3389.715	2074.307	3160.933	2113.512	1201.175	720.9281

VPA_8	no_15	IsAnnotate	NumberM	RankMS2ic	RankMS2li	RankMS2h	RankMS2k	RankMS2p
815.876	11.45197	0	0	-	-	-	-	-
1360.628	2912.413	0	0	-	-	-	-	-
3829.101	1786.17	1	0	-	-	-	-	-
3843.932	1256.334	1	0	-	-	-	-	-
5627.456	3795.558	0	0	-	-	-	-	-
2038.219	1145.227	0	0	-	-	-	-	-
1753.443	570.6018	0	0	-	-	-	-	-
821.5001	3733.728	0	0	-	-	-	-	-
1118.299	4366.797	0	0	-	-	-	-	-
629.7229	93.12334	1	0	-	-	-	-	-
1434.202	2380.318	0	0	-	-	-	-	-
2734.496	993.1383	1	0	-	-	-	-	-
2692.828	1791.157	0	0	-	-	-	-	-
8003.232	7181.32	1	0	-	-	-	-	-
1227.954	1217.818	0	0	-	-	-	-	-
103.7459	587.0502	0	0	-	-	-	-	-
2803.044	3640.087	0	0	-	-	-	-	-
2027.652	3059.074	0	0	-	-	-	-	-
1355.417	2025.429	0	0	-	-	-	-	-
431.0315	4004.91	0	0	-	-	-	-	-
579.2825	3531.185	0	0	-	-	-	-	-
7464.008	20369.8	0	0	-	-	-	-	-
2038.615	8481.837	0	0	-	-	-	-	-
158.8326	1981.95	0	0	-	-	-	-	-
25827.42	72426.99	1	0	-	-	-	-	-
27132.96	71832.25	0	0	-	-	-	-	-
21843.74	41831.54	0	0	-	-	-	-	-
5702.011	12575.06	0	0	-	-	-	-	-
1925.199	15897.13	0	0	-	-	-	-	-
3944.834	5082.063	0	0	-	-	-	-	-
3962.075	6783.727	0	0	-	-	-	-	-
7395.876	14419.08	0	0	-	-	-	-	-
6433.214	26476.21	0	0	-	-	-	-	-
8068.055	23504.86	0	0	-	-	-	-	-
6226.037	20675.71	0	0	-	-	-	-	-
19637.88	24191.53	0	0	-	-	-	-	-
4455.623	13322.7	1	0	-	-	-	-	-
4263.137	5060.611	0	0	-	-	-	-	-
1794.027	1483.875	0	0	-	-	-	-	-
1392.042	4787.997	0	0	-	-	-	-	-
3700.905	5231.695	0	0	-	-	-	-	-
20896.99	29058.59	1	0	-	-	-	-	-
28153.42	31566.01	1	0	-	-	-	-	-
994.3652	1175.062	0	0	-	-	-	-	-
10955.16	10571.48	1	0	-	-	-	-	-
5107.767	6274.267	1	0	-	-	-	-	-
248360.1	13857.4	1	0	-	-	-	-	-
2780.115	617.705	0	0	-	-	-	-	-
5990.417	11219.58	0	0	-	-	-	-	-
3041.891	4538.896	0	0	-	-	-	-	-
3405.928	1853.336	1	0	-	-	-	-	-
5274.546	7932.738	1	0	-	-	-	-	-
4284.085	14646.27	0	0	-	-	-	-	-
2382.776	4633.304	1	0	-	-	-	-	-
1375.343	1805.701	0	0	-	-	-	-	-
2200.515	707.4364	0	0	-	-	-	-	-
1220.173	627.2621	0	0	-	-	-	-	-

81064.6	288745	1	0	-	-	-	-	-
21865.76	90859.79	0	0	-	-	-	-	-
2382.859	4953.891	1	0	-	-	-	-	-
1126.112	1430.343	0	0	-	-	-	-	-
2103.372	3355.672	1	0	-	-	-	-	-
5734.586	2792.14	0	0	-	-	-	-	-
776.5753	1938.03	1	0	-	-	-	-	-
3021.478	1174.642	1	0	-	-	-	-	-
2211.943	3393.756	0	0	-	-	-	-	-
2618.962	1587.081	0	0	-	-	-	-	-
10265.19	8061.409	0	0	-	-	-	-	-
5987.331	3691.047	0	0	-	-	-	-	-
11910	3.98966	0	0	-	-	-	-	-
12532.2	29700.68	1	0	-	-	-	-	-
5629.622	10588.53	1	0	-	-	-	-	-
667.8162	3123.459	1	0	-	-	-	-	-
6536.822	1504.084	0	0	-	-	-	-	-
4831.837	10847.59	1	1	Carnitine(1	-	-	-	-
3242.226	1004.552	0	0	-	-	-	-	-
2580.623	5999.49	1	0	-	-	-	-	-
1387.745	1019.872	0	0	-	-	-	-	-
3554.886	1102.57	0	0	-	-	-	-	-
5969.054	1372.864	0	0	-	-	-	-	-
1920.981	2586.336	0	0	-	-	-	-	-
794.3309	2002.32	0	0	-	-	-	-	-
2105.145	2155.764	0	0	-	-	-	-	-
15942.58	16291.76	1	0	-	-	-	-	-
5089.604	6680.28	0	0	-	-	-	-	-
911.1667	988.9497	0	0	-	-	-	-	-
10366.74	37603.16	1	1	PC(O-16:1	LMGP0107	-	-	10814552
1444.082	633.7156	1	0	-	-	-	-	-
23452.08	63237.62	1	0	-	-	-	-	-
7492.994	15852.35	0	0	-	-	-	-	-
2534.19	1149.734	0	0	-	-	-	-	-
1096.004	1314.257	1	0	-	-	-	-	-
4980.536	5242.366	1	0	-	-	-	-	-
31296.85	11900.36	0	0	-	-	-	-	-
5437.911	13005.34	0	0	-	-	-	-	-
1641.625	3162.768	0	0	-	-	-	-	-
10787.28	36379.26	1	1	PC(O-16:1	LMGP0107	-	-	10814552
24124.71	80830.99	1	1	PC(O-16:1	LMGP0107	-	-	10814552
9087.404	25058.93	1	0	-	-	-	-	-
871.5883	1400.682	0	0	-	-	-	-	-
42743.5	18507.49	1	0	-	-	-	-	-
2454.852	8176.022	0	0	-	-	-	-	-
1914.684	3860.323	0	0	-	-	-	-	-
968651.1	3462420	1	1	PC(18:2/0:(	LMGP0105	HMDB1038	-	11005824
243712.6	905098	1	0	-	-	-	-	-
13029.03	16724.49	1	2	PC(P-16:0/	LMGP0103	-;HMDB02	-;-	24779387;:
35176.31	7223.341	0	0	-	-	-	-	-
54966.15	174678.2	1	1	PC(P-16:0/	LMGP0103	-	-	24779387
5052.82	8789.767	0	0	-	-	-	-	-
7170.897	20143.49	1	1	PE(22:6/0:(	-	-	-	-
4389.359	8064.494	1	0	-	-	-	-	-
2786.809	11571.68	1	0	-	-	-	-	-
5113.789	378.8238	0	0	-	-	-	-	-
1181.658	2660.403	0	0	-	-	-	-	-
3544.64	2901.947	0	0	-	-	-	-	-

2325.404	1772.333	0	0	-	-	-	-	-
1648.063	2262.607	1	0	-	-	-	-	-
987.655	1703.578	1	0	-	-	-	-	-
656.2098	1024.984	0	0	-	-	-	-	-
976.6452	2353.543	0	0	-	-	-	-	-
4248.331	4152.922	0	0	-	-	-	-	-
906.1486	585.6293	0	0	-	-	-	-	-
2804.951	3405.763	1	0	-	-	-	-	-
112632.1	282047.4	1	1	PC(18:2/0:	LMGP0105	HMDB103	-	11005824
32942.7	83548.41	1	0	-	-	-	-	-
138755.7	269810.7	1	3	PC(P-16:0	LMGP0103	-;HMDB10	-;-;-	24779387;
51791.98	89982.16	0	0	-	-	-	-	-
1206.17	2357.606	1	0	-	-	-	-	-
22321.61	3823.015	0	0	-	-	-	-	-
832.6423	1677.987	1	0	-	-	-	-	-
1647.947	3563.392	1	3	PC(P-18:0	LMGP0103	-;-;-	-;-;-	24779389;
3082.931	4824.284	0	0	-	-	-	-	-
4440.746	7733.283	1	0	-	-	-	-	-
2838.924	2519.503	0	0	-	-	-	-	-
10096.31	27720.88	0	0	-	-	-	-	-
1720.02	2291.2	1	0	-	-	-	-	-
2852.963	990.7257	0	0	-	-	-	-	-
1381.334	1111.861	0	0	-	-	-	-	-
92065.19	6459.068	0	0	-	-	-	-	-
9538.218	11430.5	0	0	-	-	-	-	-
16200.12	28989.66	1	1	PC(20:4/0:	LMGP0105	HMDB103	-	24779476
5585.138	10353.53	0	0	-	-	-	-	-
2013.154	4245.541	1	0	-	-	-	-	-
19672.79	64779.95	1	1	PC(22:6/0:	LMGP0105	HMDB104	-	10415542
8005.452	21991.76	1	0	-	-	-	-	-
4660.497	4383.04	0	0	-	-	-	-	-
20086.1	28697.4	1	1	PC(22:5/0:	-	-	-	-
8565.8	10409.54	0	0	-	-	-	-	-
63879.19	18766.38	1	0	-	-	-	-	-
5345.466	3930.659	0	0	-	-	-	-	-
2727.918	3109.684	0	0	-	-	-	-	-
11559.27	12493.01	0	0	-	-	-	-	-
2853.57	4333.732	1	0	-	-	-	-	-
4583.417	867.5572	1	0	-	-	-	-	-
3479.794	4805.105	1	0	-	-	-	-	-
3427.38	1777.234	1	0	-	-	-	-	-
27337.87	13461.14	1	0	-	-	-	-	-
1456.954	2391.144	0	0	-	-	-	-	-
150938	11375.42	1	0	-	-	-	-	-
35146.86	25426.25	1	0	-	-	-	-	-
386.067	1434.113	0	0	-	-	-	-	-
4408.705	2703.935	0	0	-	-	-	-	-
15651.59	6407.782	1	0	-	-	-	-	-
4361.051	9540.616	0	0	-	-	-	-	-
3001.963	13035.46	0	0	-	-	-	-	-
1206.488	1305.569	0	0	-	-	-	-	-
3154.672	14759.31	1	0	-	-	-	-	-
7392.83	24654.35	0	0	-	-	-	-	-
1511.241	2118.802	0	0	-	-	-	-	-
408487.8	18000.14	0	0	-	-	-	-	-
1392.144	1194.307	0	0	-	-	-	-	-
9308.624	40466.5	1	0	-	-	-	-	-
1648.142	1968.293	1	0	-	-	-	-	-

1777.683	1923.206	1	0	-	-	-	-	-
5105.953	4690.268	0	0	-	-	-	-	-
1722.605	423.1475	1	0	-	-	-	-	-
16246.17	20499.13	0	0	-	-	-	-	-
17418.1	8343.657	0	0	-	-	-	-	-
8977.924	6681.014	0	0	-	-	-	-	-
863.0022	1006.111	1	0	-	-	-	-	-
19237.73	3279.263	0	0	-	-	-	-	-
1403.598	4182.519	0	0	-	-	-	-	-
1106.835	1403.171	0	0	-	-	-	-	-
16067.13	19691.31	0	0	-	-	-	-	-
3925.083	4269.912	0	0	-	-	-	-	-
1389.649	1533.021	0	0	-	-	-	-	-
1442.876	1676.121	0	0	-	-	-	-	-
8468.134	16778.91	0	0	-	-	-	-	-
1009.008	1389.118	1	0	-	-	-	-	-
5285.13	1854.823	0	0	-	-	-	-	-
5272.542	16225.97	1	0	-	-	-	-	-
19373.54	15956.24	1	0	-	-	-	-	-
7679.541	6570.052	0	0	-	-	-	-	-
6742.911	19175.42	1	0	-	-	-	-	-
3317.448	7112.02	0	0	-	-	-	-	-
1271.113	3734.222	1	0	-	-	-	-	-
74126.84	62846.92	0	0	-	-	-	-	-
809.0812	2809.711	0	0	-	-	-	-	-
4503.306	6742.184	0	0	-	-	-	-	-
5592.521	13872.02	0	0	-	-	-	-	-
14755.33	22133.34	1	1	SM(d17:1/	-	-	-	-
2911.522	1660.884	0	0	-	-	-	-	-
5413.501	12321.29	0	0	-	-	-	-	-
8030.367	25232.88	1	0	-	-	-	-	-
36270.15	75490.03	1	3	PC(P-20:0/	LMGP0103	-;-;-	-;-;-	52923976;!
19524.81	6531.464	1	10	PE(18:1/16	LMGP0201	HMDB090!	-;-;-;-;-;-;-	9546802;9!
66479.19	267495.2	1	3	PC(P-20:0/	LMGP0103	-;-;-	-;-;-	52923976;!
62917.23	224.1412	0	0	-	-	-	-	-
20229.8	43924.47	1	0	-	-	-	-	-
7380.648	2456.047	0	0	-	-	-	-	-
34435.23	136523.9	1	0	-	-	-	-	-
752.3666	3269.292	0	0	-	-	-	-	-
1239.012	2265.006	0	0	-	-	-	-	-
114025	259056.3	1	0	-	-	-	-	-
49940.47	118293.2	1	0	-	-	-	-	-
13052.29	31941.45	0	0	-	-	-	-	-
10286.74	190.5577	0	0	-	-	-	-	-
11650.55	43677.7	1	0	-	-	-	-	-
5801.241	18574.58	0	0	-	-	-	-	-
1721.217	2568.402	1	0	-	-	-	-	-
217834.8	398954	1	1	SM(d18:1/	-	-	-	-
2712.474	10678.92	0	0	-	-	-	-	-
105678.2	192989.3	0	0	-	-	-	-	-
26043.86	45893.09	1	4	SM(d18:1/	LMSP0301	-;-;-;-	-;-;-;-	6453725;-;
154203.1	339854.6	1	0	-	-	-	-	-
2756.511	563.5321	1	0	-	-	-	-	-
4728.95	780.5129	1	0	-	-	-	-	-
3905.192	6213.954	0	0	-	-	-	-	-
535.1177	162.2811	0	0	-	-	-	-	-
4393.134	1687.35	0	0	-	-	-	-	-
24203.22	8363.544	1	0	-	-	-	-	-

457572.3	2273455	1	1	PC(P-16:0/	LMGP0103	HMDB112	-	24779386
27298.39	86116.93	1	1	PC(P-16:0/	LMGP0103	HMDB112	-	24779386
184293.2	903711.6	1	0	-	-	-	-	-
4653.755	2038.375	1	0	-	-	-	-	-
1575.198	1192.397	0	0	-	-	-	-	-
118823.1	400841.8	1	3	PC(P-20:0/	-;-;LMGP0	-;-;HMDB1	-;-;-	-;-;247793
43641.88	162307.3	1	0	-	-	-	-	-
229124.7	600276.9	1	3	PC(P-20:0/	LMGP0103	-;-;-	-;-;-	52923980;
103653.2	269550.4	0	0	-	-	-	-	-
30115.5	60329.08	0	0	-	-	-	-	-
6279.698	10231.15	1	0	-	-	-	-	-
2160.466	420.4799	0	0	-	-	-	-	-
6953.485	20177.12	1	0	-	-	-	-	-
7315.598	14275.3	1	0	-	-	-	-	-
7604.739	745.0682	1	0	-	-	-	-	-
19845.06	26552.9	0	0	-	-	-	-	-
2532.543	2947.695	1	0	-	-	-	-	-
7564.209	12663.63	1	0	-	-	-	-	-
26280.7	56747.19	1	0	-	-	-	-	-
2444.596	2072.413	0	0	-	-	-	-	-
7898.541	5658.85	0	0	-	-	-	-	-
384880.5	1917915	1	0	-	-	-	-	-
195004.1	1160537	1	0	-	-	-	-	-
9537.408	80.25104	1	0	-	-	-	-	-
19228.13	28323.45	1	0	-	-	-	-	-
11024.02	15791.25	1	0	-	-	-	-	-
42258.57	186177.7	0	0	-	-	-	-	-
540732.7	989620.5	1	10	PC(18:2/17	-;-;-;-;-;-;	-;-;-;-;-;-;	-;-;-;-;-;-;	-;-;-;-;-;-;
91083.54	403111.1	1	3	PC(P-20:0/	-;LMGP01	-;HMDB11	-;-;-	-;42607428
249513.1	447409.6	1	0	-	-	-	-	-
57227.81	203414.4	1	0	-	-	-	-	-
13340.71	65448.59	1	3	PC(P-20:0/	LMGP0103	-;-;-	-;-;-	52923988;
17020.69	46520.76	1	0	-	-	-	-	-
9477.107	30866.74	0	0	-	-	-	-	-
7945.21	18456.58	1	0	-	-	-	-	-
2348.921	7075.661	0	0	-	-	-	-	-
1691.858	478.17	1	0	-	-	-	-	-
1393.216	5346.294	0	0	-	-	-	-	-
3214.858	6457.058	1	0	-	-	-	-	-
5430.711	344.653	1	0	-	-	-	-	-
9048.642	17629.96	1	5	HexCer(d1	-;-;-;-;-	-;-;-;-;-	-;-;-;-;-	-;-;-;-;-
4177.844	73.6189	1	0	-	-	-	-	-
2754.7	11037.42	1	0	-	-	-	-	-
8468.843	32663.01	1	8	PC(17:1/20	-;-;-;-;-;-;	-;-;-;-;-;-;	-;-;-;-;-;-;	-;-;-;-;-;-;
146216.5	782944.1	1	2	PC(P-18:0/	-;-	-;-	-;-	-;-
14235.98	23857.9	0	0	-	-	-	-	-
1880.62	1268.457	1	0	-	-	-	-	-
98471.57	361339.8	1	0	-	-	-	-	-
8617.387	12879.93	1	0	-	-	-	-	-
16138.79	30984.5	0	0	-	-	-	-	-
10343.9	15528.03	0	0	-	-	-	-	-
5726.206	6407.061	1	0	-	-	-	-	-
46132.37	124765.2	1	3	PC(P-20:0/	-;-;-	-;-;-	-;-;-	-;-;-
124412	306609	1	2	SM(d15:1/	-;LMSP030	-;-	-;-	-;5293120
11679.72	3157.814	1	0	-	-	-	-	-
64607.65	159250.4	1	0	-	-	-	-	-
16836.09	17384.37	0	0	-	-	-	-	-
22163.84	54526.09	1	5	SM(d15:0/	-;-;-;-;LMS	-;-;-;-;-	-;-;-;-;-	-;-;-;-;529

7766.334	7813.148	1	0	-	-	-	-	-
5228.214	31906.57	1	0	-	-	-	-	-
6239.356	10224.56	0	0	-	-	-	-	-
33042.95	151055	1	8	PC(18:4/20	-;-;-;-;-;LM	-;-;-;-;-;-;-	-;-;-;-;-;-;-	-;-;-;-;-;24
1927390	5306898	1	10	PC(18:4/20	-;LMGP010	-;HMDB07	-;-;-;-;-;-;-	-;6441886;
961711.3	2334378	1	0	-	-	-	-	-
4030919	5297476	1	10	PC(18:2/20	-;-;-;-;LMC	-;-;-;-;HM	-;-;-;-;-;-;-	-;-;-;-;247
1706326	2250567	1	0	-	-	-	-	-
527760.4	1120626	1	0	-	-	-	-	-
45908.68	63953.34	1	0	-	-	-	-	-
275065.3	547166.6	1	0	-	-	-	-	-
15299.23	34996.39	0	0	-	-	-	-	-
85033.13	166161	1	2	SM(d18:1/	-;-	-;-	-;-	-;-
1127747	2328070	1	2	SM(d16:1/	-;-	-;-	-;-	-;-
12913.5	30632.05	1	0	-	-	-	-	-
583835.8	1112903	1	0	-	-	-	-	-
175293	327021.6	1	4	SM(d16:1/	-;LMSP030	-;-;-;-	-;-;-;-	-;4426012
34735.24	221950.4	1	1	PC(P-18:0/	LMGP0103	HMDB112	-	42607430
14980.79	29336.67	1	0	-	-	-	-	-
9062.06	30912.64	1	6	PC(22:4/17	-;-;-;-;LMC	-;-;-;-;-;-	-;-;-;-;-;-	-;-;-;-;247
3750.312	12681.59	1	0	-	-	-	-	-
28689.4	82404.15	1	2	SM(d15:1/	-;LMSP030	-;-	-;-	-;5293120
16425.11	46354.56	1	3	PC(P-20:0/	-;-;-	-;-;-	-;-;-	-;-;-
658.0761	42.47895	0	0	-	-	-	-	-
36658.82	99825.68	1	1	PC(P-20:0/	-	-	-	-
856.5759	20372.38	1	0	-	-	-	-	-
65839.95	102048.6	1	10	PC(22:6/16	LMGP0101	HMDB087	-;-;-;-;-;-;-	24779131;
36416.32	57583.79	1	0	-	-	-	-	-
15833.28	98425.88	1	0	-	-	-	-	-
12142.76	25661.01	1	0	-	-	-	-	-
8264.783	28901.8	1	10	PC(20:5/20	-;-;-;-;-;LM	-;-;-;-;-;HI	-;-;-;-;-;-;-	-;-;-;-;-;24
5939.063	15472.28	1	0	-	-	-	-	-
13078.7	16871.23	1	10	PC(13:0/26	-;-;LMGP0	-;-;-;-;-;-;-	-;-;-;-;-;-;-	-;-;529234
44961.85	82952.09	1	0	-	-	-	-	-
33996.83	78807.38	1	10	PC(22:2/18	-;LMGP010	-;HMDB08	-;-;-;-;-;-;-	-;2477890
24056.69	45502.5	1	0	-	-	-	-	-
120938.5	55359.95	1	0	-	-	-	-	-
14616.16	27740.01	1	2	SM(d16:1/	-;-	-;-	-;-	-;-
654.79	36.19429	0	0	-	-	-	-	-
9107.627	22455.54	1	0	-	-	-	-	-
5583.528	16561.26	1	0	-	-	-	-	-
10361.36	21684.84	0	0	-	-	-	-	-
4628.436	11464.01	1	0	-	-	-	-	-
8295.411	6303.196	0	0	-	-	-	-	-
2395.741	686.9409	1	0	-	-	-	-	-
35917.32	90270.48	1	1	PC(P-20:0/	-	-	-	-
9228.556	4468.711	1	0	-	-	-	-	-
16231.78	26782.4	1	0	-	-	-	-	-
10572.61	19480.05	1	0	-	-	-	-	-
6478.344	5837.461	1	0	-	-	-	-	-
63586.21	156.4296	0	0	-	-	-	-	-
61486.35	7324.368	0	0	-	-	-	-	-
17213.07	6174.326	1	3	TG(16:0/17	-;-;-	-;-;-	-;-;-	-;-;-
5332.413	2285.627	0	0	-	-	-	-	-
48239.81	121923.5	1	0	-	-	-	-	-
2661174	337963.4	1	3	TG(18:1/16	-;-;-	-;-;-	-;-;-	-;-;-
1830.801	2205.693	0	0	-	-	-	-	-
7914.624	11377.17	0	0	-	-	-	-	-

25828.19	65999.8	1	0	-	-	-	-	-
96366.28	11753.04	0	0	-	-	-	-	-
78077.74	66250.32	0	0	-	-	-	-	-
4550.535	5940.832	1	0	-	-	-	-	-
1884.779	2584.1	1	0	-	-	-	-	-
16812.12	6026.917	1	0	-	-	-	-	-
2100.846	171.1403	0	0	-	-	-	-	-
5317.727	3131.205	0	0	-	-	-	-	-
8019.356	2978.8	1	0	-	-	-	-	-
3934.791	12110.1	1	0	-	-	-	-	-
8339.557	23145.85	1	0	-	-	-	-	-
2199.757	2035.516	0	0	-	-	-	-	-
8725.156	11893.41	0	0	-	-	-	-	-
3300.36	2241.853	0	0	-	-	-	-	-
5678.154	5334.949	1	0	-	-	-	-	-
71634.59	73948.49	0	0	-	-	-	-	-
67678.81	71106.27	0	0	-	-	-	-	-
9712.408	14648.01	1	0	-	-	-	-	-
1247.303	943.8098	0	0	-	-	-	-	-
11203.87	4611.734	1	0	-	-	-	-	-
1109.253	591.0704	1	0	-	-	-	-	-
6708.017	1980.702	0	0	-	-	-	-	-
621.7287	2248.915	0	0	-	-	-	-	-
396.4087	4110.22	0	0	-	-	-	-	-
2794.134	6851.292	0	0	-	-	-	-	-
2504.736	4509.833	1	0	-	-	-	-	-
604.0736	24.27005	1	0	-	-	-	-	-
5751.975	12871.15	1	0	-	-	-	-	-
1763.216	5680.336	1	0	-	-	-	-	-
646.4985	97.46564	0	0	-	-	-	-	-
948.3826	687.0029	0	0	-	-	-	-	-
4594.776	10704.35	0	0	-	-	-	-	-
1468.572	4008.363	0	0	-	-	-	-	-
2807.717	3938.313	1	0	-	-	-	-	-
2713.631	8583.206	1	0	-	-	-	-	-
526.0254	3865.799	0	0	-	-	-	-	-

MS2Identi	UniqueCla	NumberM	MS1lipidm	MS1lipidm	MS1lipidm	MS1lipidmapsIdentificationLevel
-	-	0	-	-	-	-
-	-	0	-	-	-	-
-	-	52	1-(11Z-eic	LMGL0301	Triradylgly	level 2 Unique class
-	-	1	NeuAcalpl	LMSP0601	Acidic glyc	level 1 Unique lipid
-	-	0	-	-	-	-
-	-	0	-	-	-	-
-	-	0	-	-	-	-
-	-	0	-	-	-	-
-	-	0	-	-	-	-
-	-	1	NeuAcalpl	LMSP0601	Acidic glyc	level 1 Unique lipid
-	-	0	-	-	-	-
-	-	1	Delphinidi	LMPK1201	Flavonoids	level 1 Unique lipid
-	-	0	-	-	-	-
-	-	2	3'-phosph	LMFA0705	Fatty ester	level 2 Unique class
-	-	0	-	-	-	-
-	-	0	-	-	-	-
-	-	0	-	-	-	-
-	-	0	-	-	-	-
-	-	0	-	-	-	-
-	-	0	-	-	-	-
-	-	0	-	-	-	-
-	-	0	-	-	-	-
-	-	0	-	-	-	-
-	-	0	-	-	-	-
-	-	24	1'-[1-hexa	LMGP1201	Glyceroph	level 2 Unique class
-	-	0	-	-	-	-
-	-	0	-	-	-	-
-	-	0	-	-	-	-
-	-	0	-	-	-	-
-	-	0	-	-	-	-
-	-	0	-	-	-	-
-	-	0	-	-	-	-
-	-	0	-	-	-	-
-	-	0	-	-	-	-
-	-	0	-	-	-	-
-	-	0	-	-	-	-
-	-	1	sn-caldito	LMGP190	Glycerol-n	level 1 Unique lipid
-	-	0	-	-	-	-
-	-	0	-	-	-	-
-	-	0	-	-	-	-
-	-	0	-	-	-	-
-	-	2	9Z-octade	LMFA0801	Fatty amid	level 2 Unique class
-	-	2	9Z-octade	LMFA0801	Fatty amid	level 2 Unique class
-	-	0	-	-	-	-
-	-	10	3E,6Z-Hen	LMFA1200	Oxygenate	level 2 Unique class
-	-	6	6Z,9Z-Her	LMFA0500	Fatty alcoh	level 3 Multiple class
-	-	2	nonadecar	LMFA0500	Fatty alcoh	level 3 Multiple class
-	-	0	-	-	-	-
-	-	0	-	-	-	-
-	-	0	-	-	-	-
-	-	40	-;2',4'-Dih	LMPK1212	Flavonoids	level 2 Unique class
-	-	3	isopropyl	LMFA0301	Bile acids a	level 3 Multiple class
-	-	0	-	-	-	-
-	-	3	4,7,10,13-	LMFA0103	Fatty Acids	level 2 Unique class
-	-	0	-	-	-	-
-	-	0	-	-	-	-
-	-	0	-	-	-	-

-	-	7	(5Z,7E)-9,1	LMST0302	Fatty amid	level 3 Multiple class
-	-	0	-	-	-	-
-	-	2	24-nor-3a	LMST0406	Bile acids a	level 2 Unique class
-	-	0	-	-	-	-
-	-	4	(E,E)-3,7,1	LMFA0701	Fatty ester	level 3 Multiple class
-	-	0	-	-	-	-
-	-	11	-;(2R,5E,12	LMPR0105	Bile acids a	level 3 Multiple class
-	-	2	2-hydroxy	LMFA0500	Fatty Acids	level 3 Multiple class
-	-	0	-	-	-	-
-	-	0	-	-	-	-
-	-	0	-	-	-	-
-	-	0	-	-	-	-
-	-	0	-	-	-	-
-	-	9	O-9Z,12Z-	LMFA0707	Bile acids a	level 3 Multiple class
-	-	1	2R,5,7,8-te	LMPR0202	Quinones	level 1 Unique lipid
-	-	4	(4S,5aS,5b	LMPR0105	Isoprenoid	level 3 Multiple class
-	-	0	-	-	-	-
1 unique li	Fatty ester	0	-	-	-	-
-	-	0	-	-	-	-
-	-	4	25-methyl	LMFA0102	Fatty Acids	level 2 Unique class
-	-	0	-	-	-	-
-	-	0	-	-	-	-
-	-	0	-	-	-	-
-	-	0	-	-	-	-
-	-	0	-	-	-	-
-	-	0	-	-	-	-
-	-	10	3-oxo-18-	LMST0116	Fatty ester	level 3 Multiple class
-	-	0	-	-	-	-
-	-	0	-	-	-	-
1 unique li	Glyceroph	5	1-(11Z-he	LMGP0106	Glyceroph	level 2 Unique class
-	-	2	2beta,5alp	LMST0103	Bile acids a	level 3 Multiple class
-	-	13	1-(4Z,7Z,1	LMGP1005	Flavonoids	level 3 Multiple class
-	-	0	-	-	-	-
-	-	0	-	-	-	-
-	-	6	-;5,7,4'-Tri	LMPK0400	Flavonoids	level 3 Multiple class
-	-	1	1-O-alpha	LMFA1301	Fatty acyl	level 1 Unique lipid
-	-	0	-	-	-	-
-	-	0	-	-	-	-
-	-	0	-	-	-	-
1 unique li	Glyceroph	6	1-(11Z-he	LMGP0106	Fatty Acids	level 3 Multiple class
1 unique li	Glyceroph	6	1-(11Z-he	LMGP0106	Fatty Acids	level 3 Multiple class
-	-	5	(5Z,7E)-(1	LMST0302	Bile acids a	level 3 Multiple class
-	-	0	-	-	-	-
-	-	1	11R-(penta	LMFA0701	Fatty ester	level 1 Unique lipid
-	-	0	-	-	-	-
-	-	0	-	-	-	-
1 unique li	Glyceroph	2	1-(2E,4E-c	LMGP0105	Glyceroph	level 2 Unique class
-	-	6	-;(22R)-1a	LMST0101	Isoprenoid	level 3 Multiple class
2 unique li	Glyceroph	10	(2-{[(2R)-2	LMGP0105	Glyceroph	level 2 Unique class
-	-	0	-	-	-	-
1 unique li	Glyceroph	0	-	-	-	-
-	-	0	-	-	-	-
1 unique li	Glyceroph	8	(2-aminoe	LMGP0205	Glyceroph	level 2 Unique class
-	-	3	(2-{[(2R)-2	LMGP0105	Glyceroph	level 2 Unique class
-	-	4	1-nonade	LMGP0405	Glyceroph	level 3 Multiple class
-	-	0	-	-	-	-
-	-	0	-	-	-	-
-	-	0	-	-	-	-

-	-	0	-	-	-	-	
-	-	1	11R-(pent	LMFA0701	Fatty ester	level 1	Unique lipid
-	-	3	19Z,22Z,25	LMFA0103	Fatty Acids	level 3	Multiple class
-	-	0	-	-	-	-	
-	-	0	-	-	-	-	
-	-	0	-	-	-	-	
-	-	0	-	-	-	-	
-	-	1	1-(1Z-eico	LMGP0407	Glyceroph	level 1	Unique lipid
1 unique li	Glyceroph	3	1-(5Z,8Z,1	LMGP0105	Glyceroph	level 2	Unique class
-	-	5	-;(22R)-1a	LMST0101	Glyceroph	level 3	Multiple class
2 unique li	Glyceroph	13	(2-{[(2R)-2	LMGP0105	Glyceroph	level 2	Unique class
-	-	0	-	-	-	-	
-	-	2	(2-aminoe	LMGP0205	Glyceroph	level 2	Unique class
-	-	0	-	-	-	-	
-	-	1	1-(4Z,7Z,1	LMGP0105	Glyceroph	level 1	Unique lipid
2 unique li	Glyceroph	1	(5Z,7E)-(1	LMST0302	Secosteroi	level 1	Unique lipid
-	-	0	-	-	-	-	
-	-	1	1-(11Z,14Z	LMGP0405	Glyceroph	level 1	Unique lipid
-	-	0	-	-	-	-	
-	-	0	-	-	-	-	
-	-	3	N-(dodeca	LMSP0205	Ceramides	level 2	Unique class
-	-	0	-	-	-	-	
-	-	0	-	-	-	-	
-	-	0	-	-	-	-	
-	-	0	-	-	-	-	
1 unique li	Glyceroph	3	(2-{[(2R)-2	LMGP0105	Glyceroph	level 2	Unique class
-	-	0	-	-	-	-	
-	-	1	1-(4Z,7Z,1	LMGP0105	Glyceroph	level 1	Unique lipid
1 unique li	Glyceroph	3	1-(4Z,7Z,1	LMGP0105	Glyceroph	level 2	Unique class
-	-	4	(-)-6beta,1	LMPR0106	Isoprenoid	level 3	Multiple class
-	-	0	-	-	-	-	
1 unique li	Glyceroph	4	(2-{[(2R)-3	LMGP0105	Glyceroph	level 2	Unique class
-	-	0	-	-	-	-	
-	-	1	(3S,4R,9R,1	LMFA0500	Fatty alcoh	level 1	Unique lipid
-	-	0	-	-	-	-	
-	-	0	-	-	-	-	
-	-	0	-	-	-	-	
-	-	4	N-(2-hydr	LMSP0201	Ceramides	level 2	Unique class
-	-	2	-;1-trideca	LMPR0400	Diradylglyc	level 3	Multiple class
-	-	2	(2-{[(2R)-3	LMGP0105	Glyceroph	level 2	Unique class
-	-	3	-;-;-	LMPK1211	Flavonoids	level 2	Unique class
-	-	1	2S,4S,6S,8S	LMFA0102	Fatty Acids	level 1	Unique lipid
-	-	0	-	-	-	-	
-	-	2	1-(14-met	LMGL0207	Diradylglyc	level 2	Unique class
-	-	3	docosanyl	LMFA0701	Fatty ester	level 2	Unique class
-	-	0	-	-	-	-	
-	-	0	-	-	-	-	
-	-	4	13,17-Dim	LMFA1100	Hydrocarb	level 2	Unique class
-	-	0	-	-	-	-	
-	-	0	-	-	-	-	
-	-	0	-	-	-	-	
-	-	2	N-(docosa	LMSP0000	Other Sphi	level 3	Multiple class
-	-	0	-	-	-	-	
-	-	0	-	-	-	-	
-	-	0	-	-	-	-	
-	-	0	-	-	-	-	
-	-	1	1-(8-[3]-la	LMGL0203	Diradylglyc	level 1	Unique lipid
-	-	1	N-(hexade	LMSP0501	Neutral gly	level 1	Unique lipid

-	-	1	(22R)-2bet	LMST0101	Sterols [ST	level 1	Unique lipid
-	-	0	-	-	-	-	
-	-	2	-;1,3-dioct	LMGL0000	Diradylglyc	level 3	Multiple class
-	-	0	-	-	-	-	
-	-	0	-	-	-	-	
-	-	0	-	-	-	-	
-	-	3	1-(9Z,12Z,	LMGL0201	Diradylglyc	level 2	Unique class
-	-	0	-	-	-	-	
-	-	0	-	-	-	-	
-	-	0	-	-	-	-	
-	-	0	-	-	-	-	
-	-	0	-	-	-	-	
-	-	0	-	-	-	-	
-	-	0	-	-	-	-	
-	-	0	-	-	-	-	
-	-	6	1-dodecar	LMGP0401	Glyceropho	level 2	Unique class
-	-	0	-	-	-	-	
-	-	12	1-tetradec	LMGP0102	Glyceropho	level 3	Multiple class
-	-	6	1-(8Z,11Z,	LMGL0201	Diradylglyc	level 2	Unique class
-	-	0	-	-	-	-	
-	-	13	1-(1Z-hex	LMGP1003	Diradylglyc	level 3	Multiple class
-	-	0	-	-	-	-	
-	-	3	1-eicosyl-1	LMGP1002	Glyceropho	level 2	Unique class
-	-	0	-	-	-	-	
-	-	0	-	-	-	-	
-	-	0	-	-	-	-	
-	-	0	-	-	-	-	
1 unique li	Phosphosp	7	N-(15Z-te	LMSP0302	Phosphosp	level 3	Multiple class
-	-	0	-	-	-	-	
-	-	0	-	-	-	-	
-	-	4	N-(2-hydr	LMSP0302	Glyceropho	level 3	Multiple class
2 unique li	Glyceropho	17	1-tetradec	LMGP0102	Glyceropho	level 3	Multiple class
2 unique li	Glyceropho	46	1-hexadec	LMGP0101	Glyceropho	level 3	Multiple class
2 unique li	Glyceropho	17	1-tetradec	LMGP0102	Glyceropho	level 3	Multiple class
-	-	0	-	-	-	-	
-	-	2	2-methyl-	LMPR0201	Quinones a	level 3	Multiple class
-	-	0	-	-	-	-	
-	-	2	2-methyl-	LMPR0201	Quinones a	level 3	Multiple class
-	-	0	-	-	-	-	
-	-	0	-	-	-	-	
-	-	13	1-tetradec	LMGP0102	Glyceropho	level 3	Multiple class
-	-	2	cholest-5-	LMST0102	Fatty acyl g	level 3	Multiple class
-	-	0	-	-	-	-	
-	-	0	-	-	-	-	
-	-	3	N-(9Z-oct	LMSP0301	Phosphosp	level 3	Multiple class
-	-	0	-	-	-	-	
-	-	1	1-(13Z,16Z	LMGP0605	Glyceropho	level 1	Unique lipid
1 unique li	Phosphosp	10	N-(9Z-oct	LMSP0301	Phosphosp	level 3	Multiple class
-	-	0	-	-	-	-	
-	-	0	-	-	-	-	
2 unique li	Phosphosp	6	1-eicosyl-1	LMGP1002	Diradylglyc	level 3	Multiple class
-	-	1	1,2-didode	LMGL0301	Triradylgly	level 1	Unique lipid
-	-	23	1-tridecan	LMGP0101	Glyceropho	level 3	Multiple class
-	-	6	1,2-didode	LMGL0301	Triradylgly	level 2	Unique class
-	-	0	-	-	-	-	
-	-	0	-	-	-	-	
-	-	0	-	-	-	-	
-	-	25	1-octadec	LMGP1002	Glyceropho	level 3	Multiple class

1 unique li	Glycerophc	18	1-(9Z,12Z-	LMGP0109	Glycerophc	level 3	Multiple class
1 unique li	Glycerophc	18	1-(9Z,12Z-	LMGP0109	Glycerophc	level 3	Multiple class
-	-	1	N-(2R-hyc	LMSP0501	Neutral gly	level 1	Unique lipid
-	-	20	1,2-didode	LMGL0301	Neutral gly	level 3	Multiple class
-	-	0	-	-	-	-	
2 unique li	Glycerophc	17	1-(9Z-octa	LMGP0109	Glycerophc	level 3	Multiple class
-	-	1	N-(2-hydr	LMSP0501	Neutral gly	level 1	Unique lipid
2 unique li	Glycerophc	15	1-hexadec	LMGP0102	Glycerophc	level 3	Multiple class
-	-	0	-	-	-	-	
-	-	0	-	-	-	-	
-	-	2	-;N-(9Z-oc	LMPR0201	Phosphosp	level 3	Multiple class
-	-	0	-	-	-	-	
-	-	11	1-hexadec	LMGP0102	Glycerophc	level 3	Multiple class
-	-	11	1-hexadec	LMGP0102	Glycerophc	level 3	Multiple class
-	-	16	1,2-didode	LMGL0301	Triradylgly	level 2	Unique class
-	-	0	-	-	-	-	
-	-	5	N-(tricosa	LMSP0301	Phosphosp	level 2	Unique class
-	-	9	1-eicosyl-2	LMGP1002	Diradylglyc	level 3	Multiple class
-	-	14	1-(1Z-hexa	LMGP0103	Glycerophc	level 3	Multiple class
-	-	0	-	-	-	-	
-	-	0	-	-	-	-	
-	-	27	1-hexadec	LMGP0102	Glycerophc	level 3	Multiple class
-	-	2	1-hexadec	LMGP0202	Glycerophc	level 2	Unique class
-	-	1	3,7,11,15,1	LMPR0301	Polyprenol	level 1	Unique lipid
-	-	19	1-(5Z,8Z,1	LMGP1001	Glycerophc	level 3	Multiple class
-	-	13	1-(9Z-pen	LMGP0201	Glycerophc	level 3	Multiple class
-	-	0	-	-	-	-	
2 unique li	Glycerophc	42	1-tridecan	LMGP0101	Glycerophc	level 3	Multiple class
2 unique li	Glycerophc	13	1-(9Z-octa	LMGP0109	Glycerophc	level 3	Multiple class
-	-	28	1-hexadec	LMGP0101	Glycerophc	level 3	Multiple class
-	-	8	1-(1Z-octa	LMGP0103	Glycerophc	level 3	Multiple class
2 unique li	Glycerophc	0	-	-	-	-	
-	-	1	1-(6-[3]-la	LMGP0104	Glycerophc	level 1	Unique lipid
-	-	0	-	-	-	-	
-	-	14	1-eicosyl-2	LMGP1002	Glycerophc	level 3	Multiple class
-	-	0	-	-	-	-	
-	-	19	1,2-ditride	LMGL0301	Triradylgly	level 2	Unique class
-	-	0	-	-	-	-	
-	-	3	N-(pentac	LMSP0301	Phosphosp	level 2	Unique class
-	-	16	1-dodecar	LMGP0401	Glycerophc	level 2	Unique class
2 unique li	unknow	3	N-(docosa	LMSP0501	Neutral gly	level 2	Unique class
-	-	27	1,2-ditetra	LMGL0301	Triradylgly	level 2	Unique class
-	-	9	1,2-di-(4Z	LMGP1001	Glycerophc	level 3	Multiple class
2 unique li	Glycerophc	48	1-pentade	LMGP0101	Glycerophc	level 3	Multiple class
2 unique li	Glycerophc	20	1-hexadec	LMGP0102	Glycerophc	level 3	Multiple class
-	-	0	-	-	-	-	
-	-	24	1-pentade	LMGP0101	Glycerophc	level 3	Multiple class
-	-	9	1-pentade	LMGP0401	Glycerophc	level 3	Multiple class
-	-	6	1-tetradec	LMGP0102	Glycerophc	level 3	Multiple class
-	-	0	-	-	-	-	
-	-	0	-	-	-	-	
-	-	32	1-(5Z,8Z,1	LMGL0501	Glycerophc	level 3	Multiple class
2 unique li	Glycerophc	7	1-tetradec	LMGP0102	Glycerophc	level 3	Multiple class
2 unique li	Phosphosp	2	N-(15Z-te	LMSP0301	Phosphosp	level 2	Unique class
-	-	58	1-dodecar	LMGL0301	Neutral gly	level 3	Multiple class
-	-	13	1-dodecar	LMGL0301	Triradylgly	level 2	Unique class
-	-	0	-	-	-	-	
2 unique li	Phosphosp	0	-	-	-	-	

-	-	3	N-(15Z-te	LMSP0301	Phosphosp	level 2	Unique class
-	-	8	1-(1Z-eicc	LMGP1003	Glyceroph	level 3	Multiple class
-	-	0	-	-	-	-	
2 unique li	Glyceroph	50	1-(9Z-hex	LMGP0101	Glyceroph	level 3	Multiple class
2 unique li	Glyceroph	62	N-(dodeca	LMSP0501	Glyceroph	level 3	Multiple class
-	-	15	1-(1Z-eicc	LMGP1003	Glyceroph	level 3	Multiple class
2 unique li	Glyceroph	62	1-hexadec	LMGP0101	Glyceroph	level 3	Multiple class
-	-	7	1-eicosyl-:	LMGP1002	Glyceroph	level 3	Multiple class
-	-	2	N-(15Z-te	LMSP0301	Phosphosp	level 2	Unique class
-	-	1	N-(docosa	LMSP0301	Phosphosp	level 1	Unique lipid
-	-	14	1-dodecar	LMGL0301	Triradylgly	level 2	Unique class
-	-	0	-	-	-	-	
2 unique li	Phosphosp	0	-	-	-	-	
2 unique li	Phosphosp	4	N-(15Z-te	LMSP0301	Neutral gly	level 3	Multiple class
-	-	3	1-(1Z-eicc	LMGP0103	Glyceroph	level 3	Multiple class
-	-	24	1,2-ditride	LMGL0301	Triradylgly	level 2	Unique class
2 unique li	Phosphosp	0	-	-	-	-	
1 unique li	Glyceroph	15	1-(1Z-octa	LMGP0103	Glyceroph	level 2	Unique class
-	-	11	1-heptade	LMGP0401	Glyceroph	level 3	Multiple class
2 unique li	Glyceroph	42	1-heptade	LMGP0101	Glyceroph	level 3	Multiple class
-	-	2	N-(tricosa	LMSP0501	Neutral gly	level 2	Unique class
2 unique li	Phosphosp	2	N-(15Z-te	LMSP0301	Phosphosp	level 2	Unique class
2 unique li	Glyceroph	4	1-eicosyl-:	LMGP0402	Glyceroph	level 3	Multiple class
-	-	0	-	-	-	-	
1 unique li	Glyceroph	18	1-octadec	LMGP0102	Glyceroph	level 3	Multiple class
-	-	2	1-(4Z,7Z,1	LMGP0201	Glyceroph	level 2	Unique class
2 unique li	Glyceroph	40	1-(6Z,9Z,1	LMGP0101	Glyceroph	level 3	Multiple class
-	-	3	1-(5Z,8Z,1	LMGP0201	Glyceroph	level 2	Unique class
-	-	5	1-(8Z,11Z,	LMGP0201	Glyceroph	level 3	Multiple class
-	-	3	1-(1Z-eicc	LMGP0103	Glyceroph	level 3	Multiple class
2 unique li	Glyceroph	38	1-(11Z-oc	LMGP0101	Glyceroph	level 3	Multiple class
-	-	3	3-O-(6'-O	LMST0101	Sterols [ST	level 2	Unique class
2 unique li	Glyceroph	3	1-(2E,6E-p	LMGP0104	Glyceroph	level 3	Multiple class
-	-	1	N-(15Z-te	LMSP0301	Phosphosp	level 1	Unique lipid
2 unique li	Glyceroph	24	N-(tetrade	LMSP0501	Glyceroph	level 3	Multiple class
-	-	8	1-eicosyl-:	LMGP0302	Glyceroph	level 3	Multiple class
-	-	8	1-(9Z-hep	LMGP0101	Glyceroph	level 3	Multiple class
2 unique li	Phosphosp	59	1-eicosyl-:	LMGP0402	Glyceroph	level 3	Multiple class
-	-	0	-	-	-	-	
-	-	1	N-(tetraco	LMSP0301	Phosphosp	level 1	Unique lipid
-	-	32	1-(3-hydro	LMGP0404	Glyceroph	level 3	Multiple class
-	-	0	-	-	-	-	
-	-	7	1-nonadec	LMGP0401	Glyceroph	level 3	Multiple class
-	-	0	-	-	-	-	
-	-	2	Acetyl-Co/	LMFA0705	Fatty ester	level 2	Unique class
1 unique li	Glyceroph	10	1-eicosyl-:	LMGP0102	Glyceroph	level 2	Unique class
-	-	23	1-octadec	LMGP0101	Glyceroph	level 3	Multiple class
-	-	1	3-O-(6'-O	LMST0103	Sterols [ST	level 1	Unique lipid
-	-	1	3-O-(6'-O	LMST0103	Sterols [ST	level 1	Unique lipid
-	-	22	1-eicosyl-:	LMGP0402	Glyceroph	level 3	Multiple class
-	-	0	-	-	-	-	
-	-	0	-	-	-	-	
2 unique li	Triradylgly	0	-	-	-	-	
-	-	0	-	-	-	-	
-	-	7	1-tetracos	LMGP0102	Glyceroph	level 2	Unique class
2 unique li	Triradylgly	51	1-heptade	LMGL0301	Triradylgly	level 2	Unique class
-	-	0	-	-	-	-	
-	-	0	-	-	-	-	

-	-	3	1-heneicos	LMGP0401	Glyceroph	level 3	Multiple class
-	-	0	-	-	-	-	
-	-	0	-	-	-	-	
-	-	1	1-(1Z-eico	LMGP0303	Glyceroph	level 1	Unique lipid
-	-	10	1,2-diocta	LMGP0601	Glyceroph	level 3	Multiple class
-	-	66	1,2-di-(9Z	LMGL0301	Glyceroph	level 3	Multiple class
-	-	0	-	-	-	-	
-	-	0	-	-	-	-	
-	-	2	1-(11Z-do	LMGP0101	Glyceroph	level 2	Unique class
-	-	6	1-heneicos	LMGP0401	Glyceroph	level 3	Multiple class
-	-	1	1-(13Z,16Z	LMGP0102	Glyceroph	level 1	Unique lipid
-	-	0	-	-	-	-	
-	-	0	-	-	-	-	
-	-	0	-	-	-	-	
-	-	4	N-tetracos	LMSP0602	Acidic glyc	level 3	Multiple class
-	-	0	-	-	-	-	
-	-	0	-	-	-	-	
-	-	32	1-(9Z,12Z,	LMGL0301	Sterols [ST	level 3	Multiple class
-	-	0	-	-	-	-	
-	-	114	1-(9Z,12Z,	LMGL0301	Triradylgly	level 2	Unique class
-	-	5	1-nonadec	LMGP0601	Glyceroph	level 2	Unique class
-	-	0	-	-	-	-	
-	-	0	-	-	-	-	
-	-	0	-	-	-	-	
-	-	0	-	-	-	-	
-	-	4	N-(hexaco	LMSP0303	Phosphosp	level 2	Unique class
-	-	2	-;-	LMPK1211	Flavonoids	level 2	Unique class
-	-	1	2-O-hexad	LMSL0300	Acyltrehalo	level 1	Unique lipid
-	-	6	N-(15Z-te	LMSP0501	Isoprenoid	level 3	Multiple class
-	-	0	-	-	-	-	
-	-	0	-	-	-	-	
-	-	0	-	-	-	-	
-	-	0	-	-	-	-	
-	-	2	-;-	LMPR0107	Isoprenoid	level 2	Unique class
-	-	4	N-(docosa	LMSP0501	Neutral gly	level 2	Unique class
-	-	0	-	-	-	-	
